# Iron accelerates *Fusobacterium nucleatum*–induced CCL8 expression in macrophages and is associated with colorectal cancer progression

**DOI:** 10.1172/jci.insight.156802

**Published:** 2022-11-08

**Authors:** Taishi Yamane, Yohei Kanamori, Hiroshi Sawayama, Hiromu Yano, Akihiro Nita, Yudai Ohta, Hironori Hinokuma, Ayato Maeda, Akiko Iwai, Takashi Matsumoto, Mayuko Shimoda, Mayumi Niimura, Shingo Usuki, Noriko Yasuda-Yoshihara, Masato Niwa, Yoshifumi Baba, Takatsugu Ishimoto, Yoshihiro Komohara, Tomohiro Sawa, Tasuku Hirayama, Hideo Baba, Toshiro Moroishi

**Affiliations:** 1 Department of Cell Signaling and Metabolic Medicine, Faculty of Life Sciences,; 2 Department of Gastroenterological Surgery, Graduate School of Medical Sciences,; 3 Department of Cell Pathology, Graduate School of Medical Sciences, and; 4 Liaison Laboratory Research Promotion Center, Institute of Molecular Embryology and Genetics, Kumamoto University, Kumamoto, Japan.; 5 Laboratory of Pharmaceutical and Medicinal Chemistry, Gifu Pharmaceutical University, Gifu, Japan.; 6 Gastrointestinal Cancer Biology, International Research Center for Medical Sciences,; 7 Center for Metabolic Regulation of Healthy Aging, Faculty of Life Sciences, and; 8 Department of Microbiology, Graduate School of Medical Sciences, Kumamoto University, Kumamoto, Japan.

**Keywords:** Oncology, Cancer, Macrophages

## Abstract

Accumulating evidence suggests that high levels of *Fusobacterium nucleatum* in colorectal tumor tissues can be associated with poor prognosis in patients with colorectal cancer (CRC); however, data regarding distinct prognostic subgroups in *F. nucleatum*–positive CRC remain limited. Herein, we demonstrate that high-iron status was associated with a worse prognosis in patients with CRC with *F. nucleatum*. Patients with CRC presenting elevated serum transferrin saturation exhibited preferential iron deposition in macrophages in the tumor microenvironment. In addition, *F. nucleatum* induced *CCL8* expression in macrophages via the TLR4/NF-κB signaling pathway, which was inhibited by iron deficiency. Mechanistically, iron attenuated the inhibitory phosphorylation of NF-κB p65 by activating serine/threonine phosphatases, augmenting tumor-promoting chemokine production in macrophages. Our observations indicate a key role for iron in modulating the NF-κB signaling pathway and suggest its prognostic potential as a determining factor for interpatient heterogeneity in *F. nucleatum*–positive CRC.

## Graphical abstract

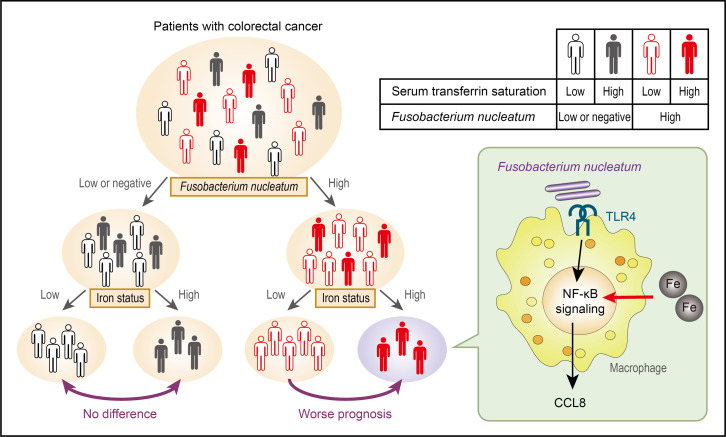

## Introduction

Colorectal cancer (CRC) is the third most commonly diagnosed cancer and the second leading cause of cancer-related deaths worldwide (1). Genetic predispositions and environmental triggers play an important role in the initiation and progression of CRC. Recent studies have shown that the gut microbiota contributes to the pathogenesis of CRC by affecting local immune responses (2). In particular, increased abundance of *Fusobacterium nucleatum* has been associated with poor prognosis in patients with CRC (3). Although it is generally believed that *F*. *nucleatum* contributes to the development of CRC by inducing chronic inflammation and suppressing the host immunity (4), some studies suggest the existence of distinct prognostic subgroups in *F*. *nucleatum*–positive CRC. For example, Oh et al. have reported a comparable prognostic effect of *F*. *nucleatum* levels in patients with stage II or III CRC treated with adjuvant chemotherapy (5), suggesting that additional factors affecting the patient background need to be considered to clarify the prognostic potential of *F*. *nucleatum* in CRC. Indeed, Yang et al. have reported that high expression of microRNA-21 can be associated with the poor prognosis of patients with CRC with high *F*. *nucleatum* levels (6). However, factors determining the interpatient heterogeneity in *F*. *nucleatum*–positive CRC need to be comprehensively elucidated.

Previous studies have revealed that *F*. *nucleatum* facilitates the development of a proinflammatory microenvironment in CRC (7). For instance, *F*. *nucleatum* was shown to recruit proinflammatory neutrophils and macrophages in a mouse model of colorectal carcinogenesis (8). Accumulated evidence has revealed that iron metabolism orchestrates the inflammatory responses to bacterial infections (9). Iron chelation reportedly upregulates the mRNA levels of *Tnf*, *Il12*, and *Ifng* in the spleens of *Salmonella typhimurium*–infected mice (10). *Mycobacterium tuberculosis*–induced nitric oxide production was found to be blunted in iron-depleted J774 macrophages (11). Furthermore, iron accumulation in macrophages was shown to promote LPS-induced TNF-α production (12). Based on these observations, we hypothesized that aberrant iron metabolism might be crucial for *F*. *nucleatum*–induced CRC progression.

Herein, we investigated the relationship between systemic iron status and CRC prognosis. We demonstrate that iron accumulation in macrophages within CRC tissues was associated with poor prognosis in *F*. *nucleatum*–positive patients with CRC. In vitro validation experiments revealed that iron was required for the efficient activation of the NF-κB signaling pathway and the consequent induction of tumor-promoting chemokines in macrophages upon *F*. *nucleatum* infection. Our results suggest a prognostic role for iron in *F*. *nucleatum*–positive CRC.

## Results

### Intratumoral iron deposition was associated with poor prognosis in patients with CRC with high F. nucleatum.

**Figure 1:**
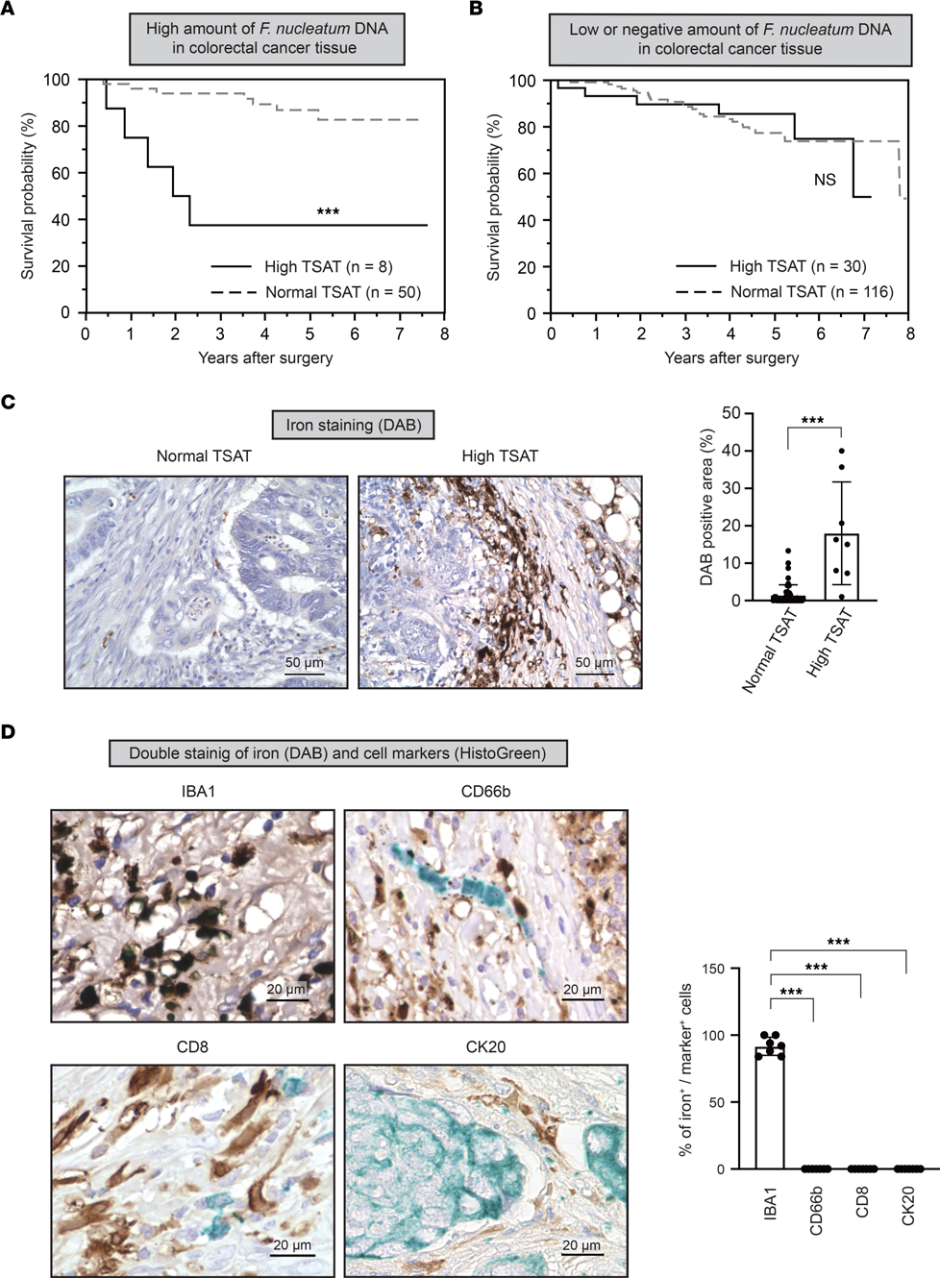
Intratumoral iron deposition is associated with poor prognosis in patients with colorectal cancer with high *F*. ***nucleatum*****levels**. (**A**) High transferrin saturation (TSAT) is associated with poor overall survival of patients with colorectal cancer (CRC) with high *F*. *nucleatum* levels. Overall survival curves for patients with CRC with high *F*. *nucleatum* levels are shown. Patients were subdivided into high (*n* = 8) or low (*n* = 50) TSAT groups. ***P < 0.001 (log-rank test). (**B**) TSAT is not associated with overall survival in patients with CRC with low or negative *F*. *nucleatum* levels. Overall survival curves for patients with CRC with low or negative levels of *F*. *nucleatum* are shown. Patients were subdivided into high (*n* = 30) or low (*n* = 116) TSAT groups. *P* > 0.05 (log-rank test). (**C**) Iron accumulates in CRC tissues in patients with high TSAT levels. DAB-enhanced Perls’ iron staining was performed on paraffin-embedded CRC tissues from patients with normal (*n* = 50) or high (*n* = 8) TSAT levels. Data are presented as the mean ± SD. ***P < 0.001 (Mann-Whitney *U* test). Scale bar: 50 μm. (**D**) Iron preferentially accumulates in macrophages within CRC tissues. Costaining of iron (DAB-enhanced Perls’ staining, shown in brown) together with immune cells (immunostaining for IBA1 [macrophage], CD66b [granulocyte], or CD8 [T cell], shown in green) or cancer cells (immunostaining for cytokeratin 20 [CK20], shown in green) was performed on paraffin-embedded CRC tissues from patients with high TSAT levels and iron deposition (*n* = 7). Scale bar: 20 μm. ***P < 0.001 (1-way ANOVA test followed by Tukey’s comparison test).

To examine the relationship between iron metabolism and *F*. *nucleatum*–induced CRC progression, patients with CRC were subdivided according to their intratumoral *F*. *nucleatum* levels and serum transferrin saturation (TSAT), an index of systemic iron status; their survival was compared using Kaplan-Meier plots. High TSAT was associated with poor overall survival (OS) in patients with CRC with high *F*. *nucleatum* levels (*P* < 0.0001) (Figure 1A and Supplemental Table 1; supplemental material available online with this article; https://doi.org/10.1172/jci.insight.156802DS1). Moreover, high TSAT was an independent risk factor for poor OS, as determined by multivariate analysis (HR, 8.33; 95% CI, 1.587–52.73; *P* = 0.012) (Table 1). In contrast, TSAT did not affect OS in patients with CRC who exhibited low and negative *F*. *nucleatum* levels (*P* = 0.93) (Figure 1B and Supplemental Table 2). These results suggest that high iron status contributes to CRC progression in the presence of *F*. *nucleatum*.

To determine the presence of iron in CRC tissues, tissue specimens from patients with CRC were subjected to iron staining. Iron deposition was observed in CRC tissues from patients with high TSAT levels, whereas only faint iron staining was observed in those with normal TSAT levels (Figure 1C). Notably, iron deposition was mostly observed in macrophages, whereas no apparent iron deposition was detected in other immune or cancer cells (Figure 1D and Supplemental Figure 1). Collectively, these results indicated that iron preferentially accumulates in macrophages within CRC tissues of patients exhibiting high iron status.

### Iron accelerated F. nucleatum–induced chemokine production in macrophages.

**Figure 2:**
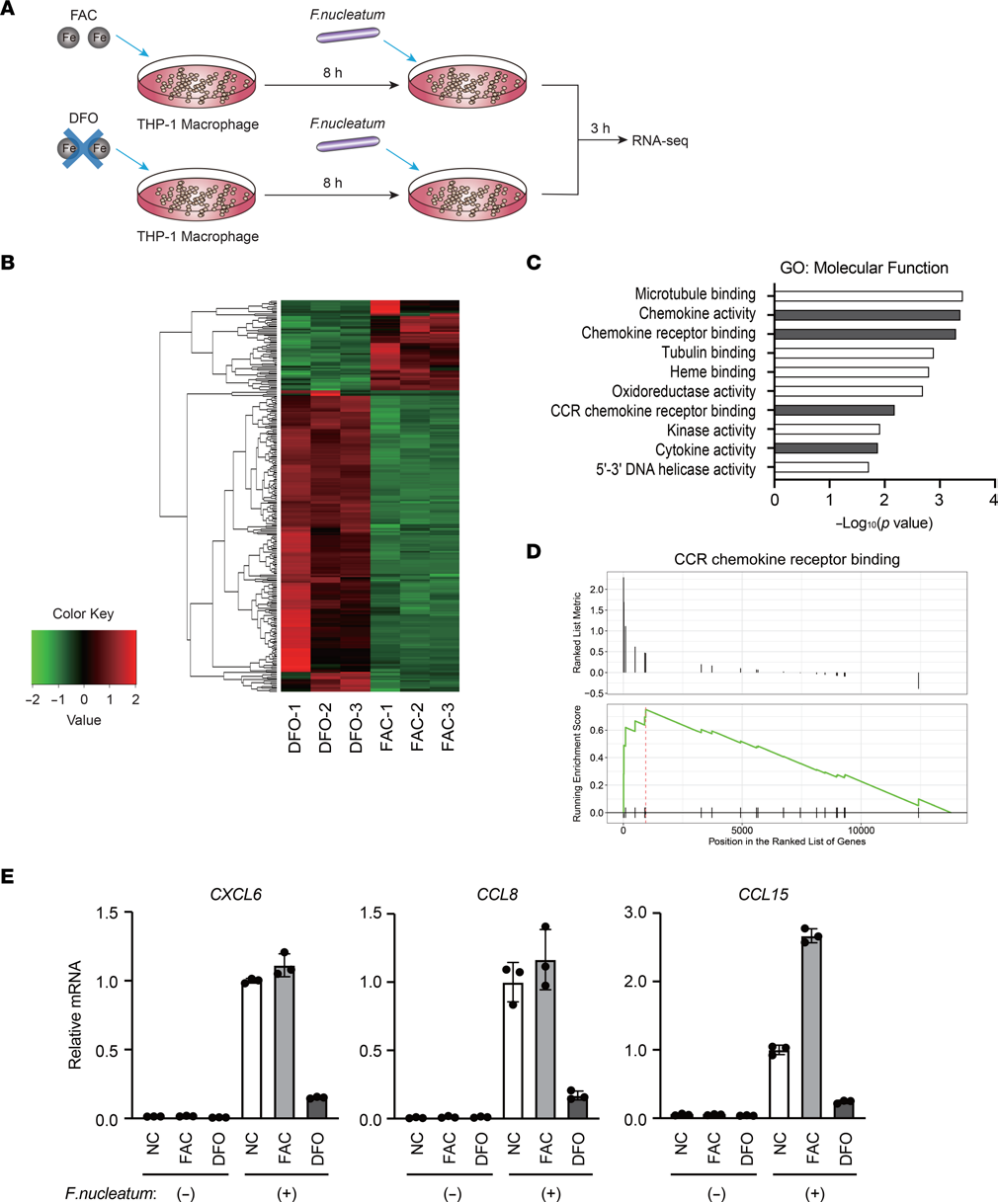
Iron accelerates *F*. ***nucleatum*****–induced chemokine expression in macrophages**. (**A**) Schematic illustration of the experimental protocol for RNA-sequencing analysis. THP-1 macrophages were pretreated with ferric ammonium citrate (FAC; 100 μM) or deferoxamine (DFO; 100 μM) for 8 hours, followed by treatment with *F*. *nucleatum* at a multiplicity of infection (MOI) of 10 for 3 hours. (**B**) Heatmap showing differentially expressed genes (fold change > 2 and adjusted *P* < 0.05) identified by RNA-sequencing analysis of THP-1 macrophages. (**C**) Genes related to chemokine signaling were enriched as the upregulated genes in FAC-treated THP-1 macrophages. Gene ontology (GO) analysis of 63 genes upregulated in FAC-treated THP-1 macrophages was performed, and the top 10 significantly enriched categories are shown. (**D**) Genes annotated with the “CCR chemokine receptor binding” GO term were upregulated in FAC-treated THP-1 macrophages. Gene set enrichment analysis of the expression pattern of genes annotated with CCR chemokine receptor binding in FAC-treated and DFO-treated THP-1 macrophages is shown. (**E**) Iron potentiates *F*. *nucleatum*–induced expression of chemokines in THP-1 macrophages. THP-1 macrophages were pretreated with FAC (100 μM) or DFO (100 μM) for 8 hours, followed by treatment with *F*. *nucleatum* at a MOI of 10 for 3 hours. RT-qPCR analysis of chemokine expression is shown. Data are presented as the mean ± SD of triplicates from a representative experiment.

Based on our initial findings, we speculated that macrophage iron metabolism is involved in the pathogenesis of *F*. *nucleatum*–induced CRC. To explore the effect of iron on macrophage properties in *F*. *nucleatum*–positive CRC tissues, THP1 human macrophages were pretreated with ferric ammonium citrate (FAC) or iron chelator deferoxamine (DFO) and stimulated with *F*. *nucleatum* (Figure 2A). RNA-sequencing analysis revealed that the expression of 63 genes was significantly increased and that of 210 genes was significantly decreased in FAC-treated cells when compared with DFO-treated cells (Figure 2B and Supplemental Table 3). Gene ontology (GO) analysis revealed that chemokine signaling–related genes were upregulated (Figure 2C and Supplemental Table 4), and metabolism-related genes were downregulated (Supplemental Figure 2 and Supplemental Table 8) in FAC-treated cells. Given that several lines of evidence indicate the important role of chronic inflammation in the pathogenesis of *F*. *nucleatum*–induced CRC (7), we focused on genes related to chemokine signaling. Gene set enrichment analysis (GSEA) revealed that genes annotated with the “CCR chemokine receptor binding” GO term were upregulated in FAC-treated cells. Among the genes annotated with “CCR chemokine receptor binding,” several chemokine genes, such as *CXCL6*, *CCL8*, and *CCL15*, were differentially increased in FAC-treated cells when compared with DFO-treated cells (Figure 2D and Supplemental Table 5). To validate our RNA-sequencing data, we further examined the expression of these chemokines in THP1 cells cocultured with *F*. *nucleatum* under iron-deficient and iron-overload conditions using reverse transcription and quantitative PCR (RT-qPCR). *F*. *nucleatum*–induced expression of these chemokines was enhanced or unaltered by iron loading, whereas it was markedly inhibited by iron depletion (Figure 2E and Supplemental Table 7). The slight difference in mRNA levels of these chemokines between FAC-treated cells and untreated cells could be attributed to the high basal levels of iron in THP-1 cells under regular cell culture conditions (Supplemental Figure 3). These observations suggest that iron is essential for the efficient production of chemokines by macrophages in response to *F*. *nucleatum*.

### F. nucleatum induced chemokine production via TLR4/NF-κB signaling, which was inhibited by iron deficiency.

**Figure 3:**
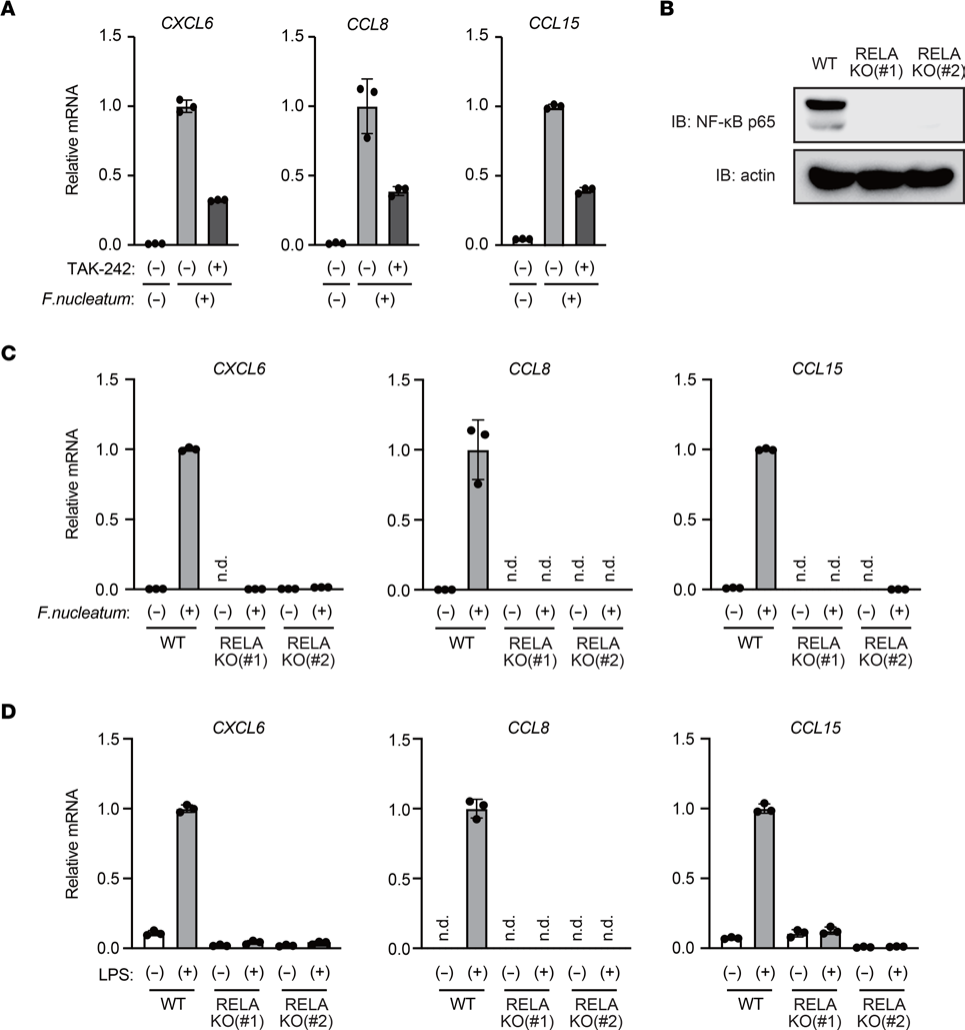
*F*. ***nucleatum*****activates TLR4/NF-κB signaling to induce chemokines in macrophages**. (**A**) TLR4 mediates *F*. *nucleatum*–induced chemokine expression in THP-1 macrophages. THP-1 macrophages were pretreated with TAK-242 (5 μM) for 1 hour, followed by treatment with *F*. *nucleatum* (MOI = 10) for 3 hours. RT-qPCR analysis of chemokine expression is shown. Data are presented as the mean ± SD of triplicates from a representative experiment. (**B**) NF-κB p65 expression was undetectable in RELA-KO THP-1 cells. Immunoblot analysis of NF-κB p65 in WT and RELA-KO THP-1 cells is shown. (**C**) NF-κB p65 is required for *F*. *nucleatum*–induced chemokine expression in THP-1 macrophages. WT and RELA-KO THP-1 macrophages were treated with *F*. *nucleatum* (MOI = 10) for 3 hours. RT-qPCR analysis of chemokine expression is shown. Data are presented as the mean ± SD of triplicates from a representative experiment. n.d., not detected. (**D**) NF-κB p65 mediates LPS-induced chemokine expression in THP-1 cells. WT and RELA-KO THP-1 cells were treated with LPS (100 ng/ml) for 3 hours. RT-qPCR analysis of chemokine expression is shown. Data are presented as the mean ± SD of triplicates from a representative experiment.

In an attempt to identify the molecular mechanisms underlying iron regulation of inflammatory chemokine production, we first investigated the signaling pathway responsible for *F*. *nucleatum*–induced chemokine expression in THP1 macrophages. *F*. *nucleatum* is a Gram-negative bacterium that contains LPS on its surface (13), and a previous study showed that the LPS-responsive TLR4/NF-κB pathway is activated in response to *F*. *nucleatum* (6). Treatment with TAK-242, a TLR4 inhibitor, suppressed *F*. *nucleatum*–induced gene expression of *CXCL6*, *CCL8*, and *CCL15* in THP1 macrophages (Figure 3A). Moreover, *F*. *nucleatum*–induced upregulation of these genes was abrogated in RELA-KO (human *RELA* gene encodes NF-κB p65 protein) THP-1 cells when compared with WT cells (Figure 3, B and C; Supplemental Figure 4, A–C; and Supplemental Tables 6 and 9). Additionally, we confirmed that expression of these chemokines can be induced by LPS treatment in THP1 cells; this expression was almost completely inhibited in RELA-KO THP-1 cells (Figure 3D). Taken together, our observations indicated that TLR4/NF-κB signaling is required for *F*. *nucleatum*–induced gene expression of *CXCL6*, *CCL8*, and *CCL15* in THP-1 macrophages.

**Figure 4:**
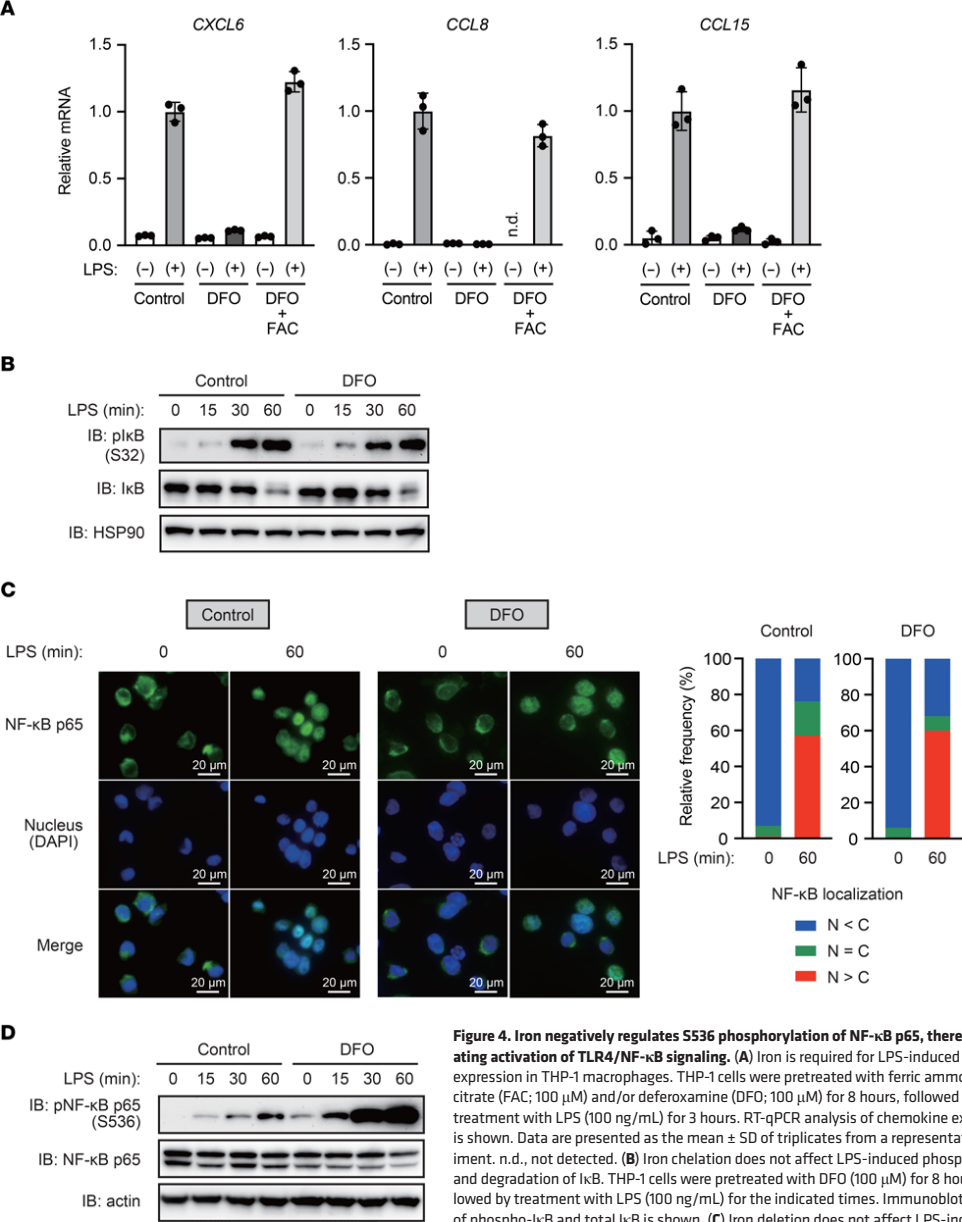
Iron negatively regulates S536 phosphorylation of NF-κB p65, thereby accelerating activation of TLR4/NF-κB signaling. (**A**) Iron is required for LPS-induced chemokine expression in THP-1 macrophages. THP-1 cells were pretreated with ferric ammonium citrate (FAC; 100 μM) and/or deferoxamine (DFO; 100 μM) for 8 hours, followed by treatment with LPS (100 ng/mL) for 3 hours. RT-qPCR analysis of chemokine expression is shown. Data are presented as the mean ± SD of triplicates from a representative experiment. n.d., not detected. (**B**) Iron chelation does not affect LPS-induced phosphorylation and degradation of IκB. THP-1 cells were pretreated with DFO (100 μM) for 8 hours, followed by treatment with LPS (100 ng/mL) for the indicated times. Immunoblot analysis of phospho-IκB and total IκB is shown. (**C**) Iron deletion does not affect LPS-induced nuclear translocation of NF-κB p65. THP-1 cells were pretreated with DFO (100 μM) for 8 hours, followed by treatment with LPS (100 ng/mL) for the indicated times. Immunofluorescence staining for NF-κB p65 is shown. N, nuclear; C, cytoplasmic. Scale bar: 20 μm. (**D**) Iron deficiency enhances the inhibitory phosphorylation of NF-κB p65 at S536. THP-1 cells were treated with DFO (100 μM) for 8 hours prior to LPS (100 ng/mL) treatment for the indicated time, followed by immunoblot analysis.

As described above, given that DFO treatment is suitable for effectively evaluating the roles of intracellular iron in THP-1 cells under cell culture conditions (Supplemental Figure 3), we examined the effects of iron deficiency to explore the link between iron and TLR4/NF-κB signaling in the following experiments. DFO inhibited LPS-induced upregulation of *CXCL6*, *CCL8*, and *CCL15* (Figure 4A). Supplementation of media with FAC almost completely reversed the suppressive effect of DFO, suggesting that iron chelation is responsible for the suppressive effects of DFO on LPS-induced chemokine production. We also observed that LPS-induced chemokine gene expression was inhibited by another iron chelator (Supplemental Figure 5). These data indicated that iron is involved in the efficient induction of chemokine genes under TLR4 signaling.

Activation of TLR4 reportedly induces phosphorylation of inhibitor of κB (IκB) by the IκB kinase (IKK) complex and degradation of IκB (14). NF-κB released from IκB translocates into the nucleus and induces the expression of its target genes. Herein, we observed that treatment with DFO did not affect the levels of phospho-IκB and total IκB, nor did nuclear translocation of NF-κB p65 in LPS-stimulated THP1 cells (Figure 4, B and C), suggesting that cellular iron status does not affect LPS-induced nuclear translocation of NF-κB in THP-1 cells. In contrast, we found that DFO markedly increased LPS-induced phosphorylation of NF-κB p65 at serine 536, which reportedly suppresses the transcriptional activity of NF-κB (15) (Figure 4D). These results suggest that iron negatively regulates the inhibitory phosphorylation of NF-κB p65 at S536, thereby enhancing TLR4/NF-κB signaling.

### Iron deficiency induced the inhibitory phosphorylation of NF-κB p65 by inhibiting protein phosphatases.

We hypothesized that two potential mechanisms underlie the enhanced inhibitory phosphorylation of NF-κB p65 upon iron depletion. First, kinases that promote NF-κB p65 phosphorylation at S536 may be suppressed by iron. Second, phosphatases that remove S536 phosphorylation may be activated by iron.

We first aimed to examine the association between iron and kinases responsible for the phosphorylation of NF-κB p65 at S536. The phosphorylation of NF-κB p65 at S536 is mediated by the IKK complex (14). The IKK complex consists of 2 catalytic subunits (IKKα and IKKβ) and a regulatory subunit (IKKγ), integrating signals from upstream NF-κB activating stimuli to catalyze the phosphorylation of various substrates, including NF-κB p65 and IκB.

**Figure 5:**
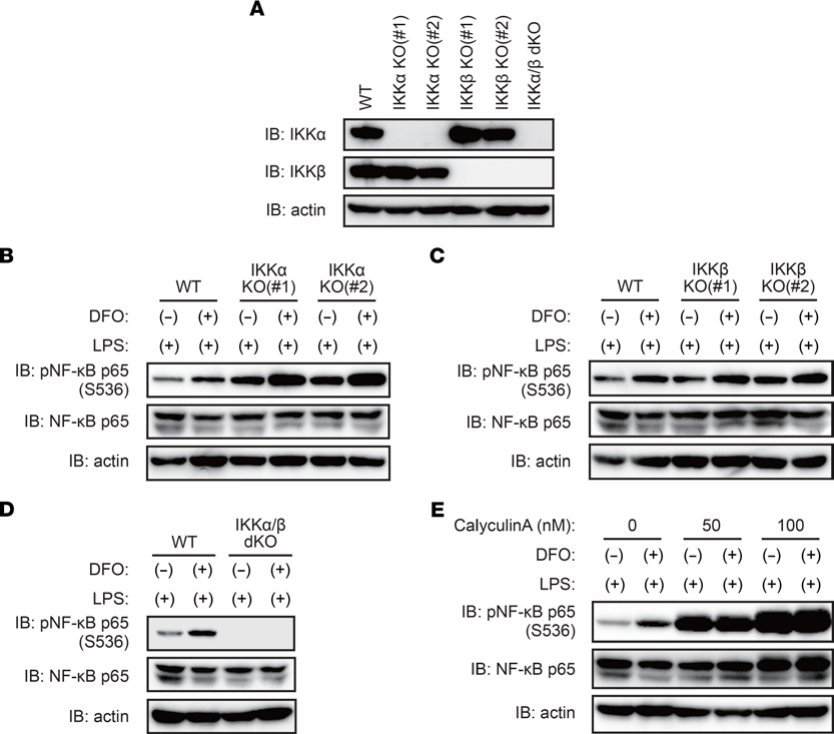
Protein phosphatases PP1 and PP2A require iron to limit inhibitory phosphorylation of NF-κB p65 upon TLR4 activation. (**A**) IKKα/β expression was undetectable in IKK-KO THP-1 cells. Immunoblot analysis of IKKα and IKKβ in WT, IKK-KO, IKKβ-KO and IKKα/β double-KO (dKO) THP-1 cells is shown. (**B**) Deletion of IKKα did not suppress iron chelation–induced inhibitory phosphorylation of NF-κB p65 at S536. WT and IKKα-KO THP-1 cells were pretreated with deferoxamine (DFO; 100 μM) for 8 hours, followed by treatment with LPS (100 ng/mL) for 1 hour. Immunoblot analysis of NF-κB p65 phosphorylation at S536 is shown. (**C**) Deletion of IKKβ has no effect on iron chelation–induced inhibitory phosphorylation of NF-κB p65 at S536. WT and IKK-KO THP-1 cells were treated with DFO (100 μM) for 8 hours prior to LPS (100 ng/mL) treatment for 1 hour and then subjected to immunoblot analysis. (**D**) Dual inhibition of IKKα and IKKβ blocks S536 phosphorylation of NF-κB p65. WT and IKKα/β dKO THP-1 cells were treated with DFO (100 μM) for 8 hours prior to LPS (100 ng/mL) treatment for 1 hour, followed by immunoblot analysis. (**E**) Iron deficiency enhances the inhibitory phosphorylation of NF-κB p65 by inhibiting the protein phosphatase PP1/PP2A. THP-1 cells were treated with DFO (100 μM) for 8 hours and stimulated with LPS (100 ng/mL) for 1 hour in the presence or absence of calyculin A. Immunoblot analysis of NF-κB p65 phosphorylation at S536 is shown.

A previous study has revealed that S536 phosphorylation of NF-κB p65 relies on IKKα in murine macrophages (16), whereas IκB phosphorylation relies on IKKβ (17). As DFO treatment did not affect LPS-induced IκB phosphorylation (Figure 4B), we postulated that iron could selectively inhibit IKKα-mediated phosphorylation of NF-κB p65 at S536. However, in our experimental settings, IKKα deletion did not decrease S536 phosphorylation of NF-κB p65 in response to LPS, irrespective of DFO treatment in THP-1 cells (Figure 5, A and B; Supplemental Figure 6, A and B, and Supplemental Tables 6 and 9). Furthermore, we detected no apparent difference in S536 phosphorylation between IKKβ-KO cells and WT cells (Figure 5, A and C; Supplemental Figure 7; and Supplemental Tables 6 and 9). In contrast, S536 phosphorylation of NF-κB p65 was largely undetectable in IKKα/β double-KO THP-1 cells, even under LPS treatment conditions (Figure 5, A and D; Supplemental Figure 8; and Supplemental Tables 6 and 9). These results indicated that (a) the IKK complex is necessary for S536 phosphorylation of NF-κB p65 under TLR4 activation and (b) the 2 catalytic subunits IKKα and IKKβ are redundant in terms of their ability to induce S536 phosphorylation of NF-κB p65 upon TLR4 activation. Thus, these observations do not support our initial hypothesis that iron inhibits IKKα-dependent phosphorylation of NF-κB p65 at S536.

We next investigated the association between iron and phosphatases responsible for the dephosphorylation of NF-κB p65 at S536. Serine/threonine phosphatases PP1 and PP2A have been shown to dephosphorylate S536 of NF-κB p65 (18, 19). We observed that pretreatment with calyculin A, an inhibitor of PP1 and PP2A, increased S536 phosphorylation of NF-κB p65 in LPS-stimulated THP1 cells in a dose-dependent manner. Importantly, there was no apparent difference in the levels of S536-phosphorylated NF-κB p65 between DFO-treated and untreated cells in the presence of calyculin A, suggesting that iron deficiency inhibits PP1 and/or PP2A; the result of this is the promotion of S536 phosphorylation of NF-κB p65 (Figure 5E). These results indicated that iron-dependent phosphatases PP1 and/or PP2A limit the levels of inhibitory phosphorylation of NF-κB p65 at S536, resulting in the maximal activation of TLR4/NF-κB signaling under iron-rich conditions.

### High expression of CCL8 was associated with poor prognosis in patients with CRC.

**Figure 6:**
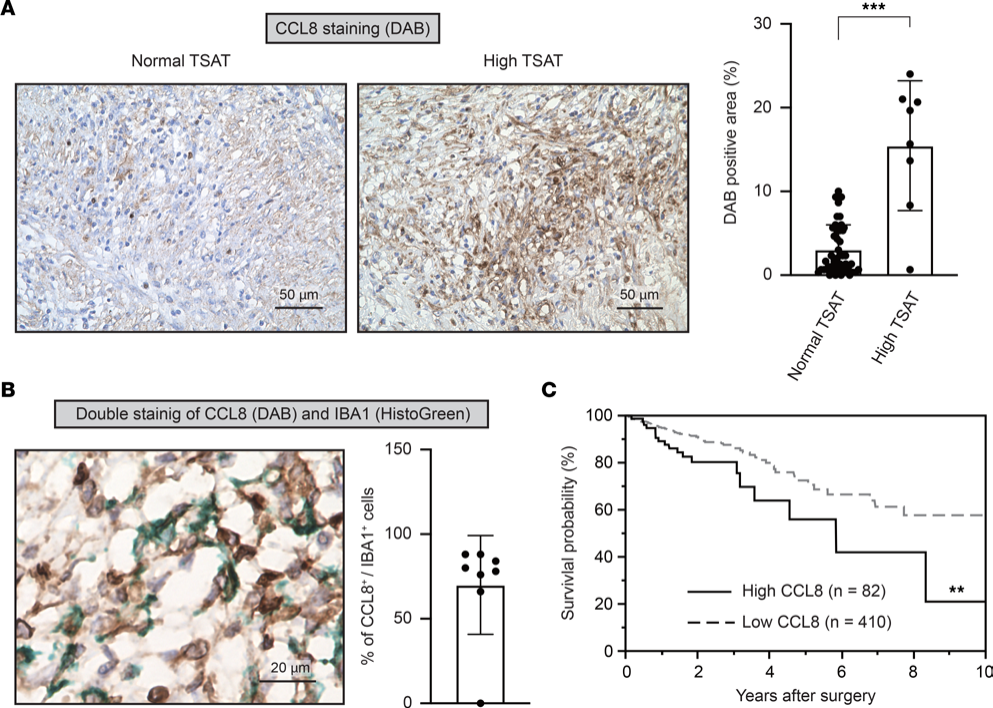
High expression of *CCL8* is associated with poor prognosis in patients with colorectal cancer. (**A**) CCL8 is highly expressed in colorectal cancer (CRC) tissues from patients with high TSAT levels. CCL8 staining was performed on paraffin-embedded CRC tissues from patients with normal (*n* = 50) or high (*n* = 8) TSAT levels. Scale bar: 50 μm. Data are presented as the mean ± SD. ***P < 0.001 (Mann-Whitney *U* test). (**B**) Macrophages produce CCL8 within CRC tissues. Costaining of CCL8 (shown in brown) and IBA1 (shown in green) was performed on paraffin-embedded CRC tissues from patients with high TSAT levels (*n* = 8). Scale bar: 20 μm. Data are presented as the mean ± SD. (**C**) Overall survival curves for patients with stage I–III CRC from The Cancer Genome Atlas data set with high expression of *CCL8* (*n* = 82) or low expression of *CCL8* (*n* = 410) are shown. The cutoff value of FPKM was 2.5. **P < 0.01 (log-rank test).

Our in vitro data suggest that iron accelerates *F*. *nucleatum*–induced inflammatory chemokine expression, including CXCL6, CCL8, and CCL15, via TLR4/NF-κB signaling in macrophages. Among these chemokines, CCL8 is mainly secreted by macrophages in the tumor microenvironment (20–22), whereas cancer cells, rather than macrophages, are the primary source of CXCL6 and CCL15 (23, 24). Indeed, the expression level of CCL8 was significantly higher in patients with *F*. *nucleatum*–positive CRC with high TSAT levels than in those with normal TSAT levels (Figure 6A). Coimmunostaining analysis using CCL8 and IBA1 antibodies revealed that macrophages produce CCL8 in patients with *F*. *nucleatum*–positive CRC with high TSAT levels (Figure 6B). To examine the clinical relevance of CCL8 chemokines in CRC progression, we examined *CCL8* mRNA expression in patients with stage I–III CRC from The Cancer Genome Atlas database. Kaplan-Meier survival analysis revealed that high *CCL8* expression was associated with poor OS in patients with CRC (*P* = 0.0077) (Figure 6C). Taken together, these results suggest that macrophage-derived CCL8 is associated with poor prognosis in patients with *F*. *nucleatum*–positive CRC with high iron status.

## Discussion

CCL8 belongs to the CC chemokine subfamily (25) and plays a pivotal role in various diseases, such as human immunodeficiency virus–associated dementia and visceral hypersensitivity induced by inflammatory bowel disease and colitis (26–28). Previous observations suggest the involvement of macrophage-derived CCL8 in the migration and invasion of cancer cells, including glioblastoma and squamous cell carcinoma cells (21, 22, 29). In the present study, we demonstrated that high expression levels of *CCL8* mRNA in tumor tissues were associated with poor prognosis of patients with stage I–III CRC (Figure 6C), implying that CCL8 may be a useful prognostic biomarker for patients with CRC. In addition, we observed that *F*. *nucleatum* induces *CCL8* expression in macrophages via the TLR4/NF-κB signaling pathway. Our data indicate that NF-κB is the main transcription factor involved in *F*. *nucleatum*–induced *CCL8* expression in macrophages, as RELA deletion markedly suppresses its induction (Figures 3C). This finding is consistent with those in a recent report that used a computational approach to predict CCL8 as a putative NF-κB target in prostate cancer (30). Moreover, our observations, together with previous findings (31, 32), established a key role for iron in modulating the NF-κB signaling pathway. It has been reported that iron potentiates NF-κB signaling by upregulating the kinase activity of IKK, resulting in phosphorylation and degradation of IκB and consequent NF-κB activation in Kupffer cells (31, 32). However, in the present study, iron deprivation did not affect the phosphorylation status or total abundance of IκB under TLR4 activation (Figure 4B). Although the precise molecular mechanisms underlying these controversial results remain elusive, the regulatory mechanism of NF-κB activation by cellular iron might be cell-type dependent, conferring the stimulatory effects of iron on the NF-κB signaling pathway via multiple pathways.

The dynamics of reversible protein phosphorylation are maintained by a balance between kinases and phosphatases (33). Although the regulatory mechanisms of protein kinases have been intensively studied, the importance and dynamic regulation of protein phosphatases are poorly explored. Previous studies have revealed that protein phosphatase activity is modulated by multiple factors, including polyamines and metal ions (34, 35). The protein serine/threonine phosphatase PP2A isolated from rabbit skeletal muscle can be directly activated by ferrous iron and ascorbate in vitro (36), suggesting that cellular ferrous iron may serve as a biological cofactor for PP2A activity. Consistently, we demonstrated that cellular iron availability is crucial for the meditation of the functions of phosphatases PP1 and/or PP2A in macrophages (Figure 5E), contributing to the regulation of inflammatory responses of macrophages upon *F*. *nucleatum* infection.

Several lines of evidence indicate that excess iron is associated with poor patient outcomes in various types of cancers, including CRC (37). For instance, dietary iron overload exacerbates colonic inflammation and promotes tumor development in mouse models of inflammation-associated colorectal tumorigenesis (38). Another study has demonstrated that preoperative iron status predicts the prognosis of patients with stage II and III CRC (39). In the current study, we revealed that high iron status is associated with a worse prognosis in patients with CRC infected with *F*. *nucleatum*. Collectively, these findings not only demonstrate a key role for iron in colorectal tumorigenesis, but also reveal its potential as a determinant factor for interpatient heterogeneity in *F*. *nucleatum*–positive CRC. Elucidating the molecular and cellular mechanisms underlying the effects of iron on chronic inflammation and tumor progression in *F*. *nucleatum* infection may open new avenues for the future development of precision prevention and valuable therapy for *F*. *nucleatum*–positive CRC.

## Methods

Further information can be found in Supplemental Methods.

### Patients.

From January 2005 to December 2019, a consecutive series of 546 patients underwent elective colorectal resection for pathological stage I–III CRC at Kumamoto University Hospital. Of these, patients were excluded if they met any one of the following criteria: (a) fresh frozen CRC tissue was not available (266 patients); (b) preoperative TSAT was not available (76 patients). Finally, 204 patients were retrospectively analyzed in this study.

### DNA extraction and qPCR for intratumor F. nucleatum.

Genomic DNA was isolated from frozen CRC tissues, and quantitative PCR was performed to measure the amount of tissue DNA that was positive for *F*. *nucleatum* as previously described (40). Cases with detectable *F*. *nucleatum* DNA were categorized as high or low according to the median cutoff for *F*. *nucleatum* DNA; cases with undetectable *F*. *nucleatum* DNA were defined as negative.

### Evaluation of TSAT and patient characteristics.

We evaluated the preoperative iron status using TSAT, calculated as the ratio of serum iron to total iron-binding capacity (41). We defined TSAT as ≥30%, which is higher than the normal range, as high levels of TSAT and TSAT <30% were considered normal.

We subdivided enrolled patients according to their intratumoral *F*. *nucleatum* levels and preoperative TSAT levels. Patient characteristics according to TSAT levels in groups with high amounts of *F*. *nucleatum* are shown in Supplemental Table 1, while characteristics of the low and negative *F*. *nucleatum* groups are shown in Supplemental Table 2.

### Histological analysis.

CRC tissues were fixed using neutral-buffered formalin and embedded in paraffin. The sections were stained with Perls’ reagent and developed using DAB, as previously described (42). After iron staining, the slides were subsequently incubated with primary antibodies against CD8 (clone SP16, ab9829; Abcam), CD66b (clone G10F5, 555723; BD Pharmingen), cytokeratin 20 (clone Ks20.8, 413491; Nichirei), and IBA1 (polyclonal, 019-19741; Wako) overnight at 4°C. The sections were visualized using HistoGreen (E109; Cosmo Bio) and counterstained with Mayer hematoxylin. CCL8 (clone 1.1_2D4-1A3, LS-B8198; LSBio) staining was conducted using DAB, followed by costaining with IBA1 using HistoGreen. Images were obtained with a KEYENCE BZ-X800 all-in-one microscope (KEYENCE). Quantification was performed using the KEYENCE BZ analyzer.

### Cell culture.

Briefly, THP-1 human monocytes (TIB-202; ATCC) were cultured in RPMI-1640 (189-02025; Wako) supplemented with 10% fetal bovine serum (175012; Nichirei), penicillin (100 U/mL) (168-23191; Wako), and streptomycin (100 mg/mL) (168-23191; Wako). *F*. *nucleatum* was cultured as previously described (43). THP-1 monocytes were pretreated with 100 μM FAC (RES20400-A7; Sigma-Aldrich), 100 μM DFO (D9533; Sigma-Aldrich), or the indicated concentration of calyculin A (038-14453; Wako) for the indicated time, followed by stimulation with 100 ng/mL LPS (tlrl-eblps; InvivoGen) for the indicated time. THP-1 monocytes were differentiated into macrophages using 6 ng/mL phorbol 12-myristate 13-acetate (AG-CN2-0010; AdipoGen) for 48 hours. Differentiated cells were pretreated with 100 μM FAC, 100 μM DFO, or 5 μM TAK-242 (13871; CAYMAN) for the indicated times, followed by treatment with *F*. *nucleatum* at a multiplicity of infection of 10 for 3 hours.

### RNA sequencing.

Total RNA was extracted from THP1 cells using TRIzol reagent (15596026; Invitrogen). Libraries were constructed from total RNA using the TruSeq Stranded mRNA Library Prep kit (RS-122-2101; Illumina). The quality of the libraries was determined using an Agilent 2200 TapeStation (Agilent Technologies). High-throughput sequencing was performed using a NextSeq500 instrument (Illumina). The reads were mapped against the human (hg38) genome using HISAT2. Differential expression analysis was performed using the DESeq2. Differentially expressed genes were selected according to fold change and adjusted *P* value (fold change > 2, adjusted *P* < 0.05, Supplemental Table 3). GO analysis of differentially expressed genes was performed using the Enrichr platform (https://maayanlab.cloud/Enrichr/; Supplemental Table 4). Fold change rankings were used in GSEA (http://software.broadinstitute.org/gsea/index.jsp) to identify differentially regulated pathways (*q* < 0.05; Supplemental Table 5).

### Gene deletion using CRISPR/Cas9 system.

RELA, IKKα, IKKβ-KO THP-1 cells were generated by the CRISPR/Cas9 system (44). The CRISPR/Cas9 guide RNA targeting guide sequences were designed using the CRISPR (http://crispor.tefor.net/). The guide sequences are listed in Supplemental Table 6. Targeting sequences were cloned into the lentiCRISPR v2 plasmid (52961; Addgene). The lentiviral transfer plasmids were cotransfected with the packaging plasmids pLenti-P2A and pLenti-P2B (LV003; Applied Biological Materials Inc.) into HEK293T cells using PolyJet (SL100688; SignaGen). Forty-eight hours after transfection, lentiviral supernatant was supplemented with 5 μg/mL polybrene (TR-1003-G; MERCK), filtered through a 0.45 μm filter (IPVH00010; MERCK), and used for infection. Forty-eight hours after infection, THP-1 cells were selected with puromycin (A1113803; Thermo Fisher Scientific) in culture medium. Subsequently, the cells were single-cell sorted using fluorescence-activated cell sorting. We selected KO clones by immunoblot analysis.

### RT-qPCR analysis.

Total RNA was extracted from cells using TRIzol (15596026; Invitrogen), and cDNA was synthesized using the ReverTra Ace qPCR RT kit (FSQ-301; TOYOBO), according to the manufacturer’s protocols. Quantitative PCR was performed using Luna Universal qPCR Master Mix (M3003; New England Biolabs). The sequences of qPCR primers are listed in Supplemental Table 7. Data were normalized to *GAPDH* levels and analyzed using the ΔΔCT method. We defined a CT value of more than 40 or nonspecific amplification in the melt curve analysis as not detected.

### Immunoblot analysis.

In brief, equal amounts of protein samples were resolved using SDS-PAGE under reducing conditions. The following antibodies were purchased and used in the present study: antibodies against IKKα (catalog 11930), IKKβ (catalog 8943), IκB (catalog 4814), pIκB (S32; 2 catalog 859), NF-κB p65 (catalog 8242), and pNF-κB p65 (S536; catalog 3033) were procured from Cell Signaling Technology; antibodies against actin (ab3280) were from Abcam; antibodies against HSP90 (catalog 610418) were purchased from BD Biosciences. See complete unedited blots in the supplemental material (Supplemental Figures 9 and 10).

### Immunofluorescent staining.

Briefly, cells were fixed with 4% paraformaldehyde and permeabilized with 0.1% Triton X-100. Then, cells were stained with anti–NF-κB p65 antibody (8242; Cell Signaling Technology) and Alexa Fluor 488–labeled goat secondary antibody (A-11008; Invitrogen). Images were obtained with the KEYENCE BZ-X800, and quantification was performed using a BZ analyzer (KEYENCE).

### Analysis of published data.

OS was compared among patients with stage I–III CRC presenting high or low levels of *CCL8* expression in The Cancer Genome Atlas data set accessed with the Human Protein Atlas website (45). Downloaded clinical data (follow-up period and dead or alive) of patients with stage I–III CRC were matched to the *CCL8* expression (FPKM value) in tumor tissues and analyzed using the Kaplan-Meier survival method.

### Data availability statement.

The data generated in the present study are publicly available in the Gene Expression Omnibus (GSE192400).

### Statistics.

Statistical analyses were performed using JMP version 13.1 software (SAS Institute). Quantitative data are presented as the mean ± SD unless indicated otherwise. Comparisons between 2 groups were performed using the Mann-Whitney *U* test. Comparisons among 3 groups were performed using 1-way ANOVA, followed by Tukey’s multiple-comparison test. Differences between survival curves were analyzed using the log-rank nonparametric test. Logistic regression analysis was performed to estimate the HR with a 95% CI for OS. Statistical significance was set at *P* < 0.05.

### Study approval.

Written informed consent was obtained from each participant, and the study procedures were approved by the Institutional Review Board of Kumamoto University (registry 1272).

## Author contributions

TY conceptualized the study; curated data; provided formal analysis, investigation, validation, and visualization; and wrote the original draft and reviewed and edited the manuscript. Y Kanamori curated data; provided formal analysis, investigation, project administration, supervision, and visualization; and wrote the original draft and reviewed and edited the manuscript. HS curated data, provided investigation, and reviewed and edited the manuscript. HY provided investigation and validation and reviewed and edited the manuscript. AN provided methodology and investigation and reviewed and edited the manuscript. YO provided investigation and reviewed and edited the manuscript. HH provided investigation and reviewed and edited the manuscript. AM provided methodology and investigation and reviewed and edited the manuscript. AI provided investigation and validation and reviewed and edited the manuscript. TM provided investigation and validation and reviewed and edited the manuscript. MS provided methodology and reviewed and edited the manuscript. MN provided investigation and reviewed and edited the manuscript. SU curated data; provided investigation and validation; and reviewed and edited the manuscript. NYY provided methodology and investigation and reviewed and edited the manuscript. MN provided methodology and reviewed and edited the manuscript. YB provided methodology; acquired funding; and reviewed and edited the manuscript. TI provided methodology and reviewed and edited the manuscript. Y Komohara provided methodology, investigation, and validation and reviewed and edited the manuscript. TS provided methodology and resources and reviewed and edited the manuscript. TH provided methodology and reviewed and edited the manuscript. HB provided project administration, resources, and supervision and reviewed and edited the manuscript. TM conceptualized the study; provided formal analysis; acquired funding; provided methodology, project administration, resources, supervision, and visualization; and wrote the original draft and reviewed and edited the manuscript.

## Supplementary Material

Supplemental data

Supplemental table 1

Supplemental table 2

Supplemental table 3

Supplemental table 4

Supplemental table 5

Supplemental table 6

Supplemental table 7

Supplemental table 8

Supplemental table 9

## Figures and Tables

**Table 1:**
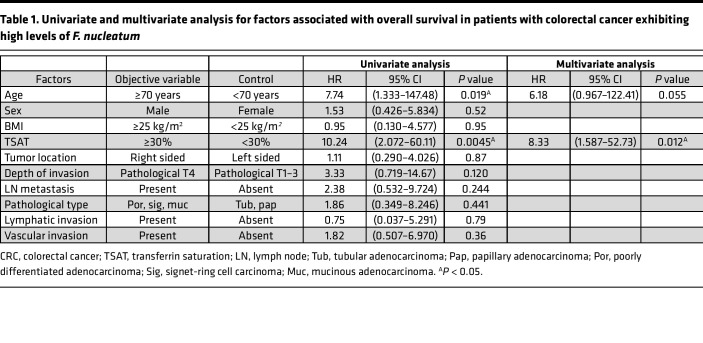
Univariate and multivariate analysis for factors associated with overall survival in patients with colorectal cancer exhibiting high levels of *F. nucleatum*

## Version 1 09/22/2022

In-Press Preview

## Version 2 11/08/2022

Electronic publication
