# Supplementary material for: Iron accelerates Fusobacterium nucleatum–induced CCL8 expression in macrophages and is associated with colorectal cancer progression
Source: JCI Insight. 2022 Nov 8;7(21):e156802. doi: 10.1172/jci.insight.156802 (PMC9675438; doi:10.1172/jci.insight.156802)
Supplement: Supplemental data [file jciinsight-7-156802-s101.pdf]

SUPPLEMENTARY INFORMATION

Iron accelerates *Fusobacterium nucleatum*-induced CCL8 expression in macrophages and is associated with colorectal cancer progression

Taishi Yamane,[1,2] Yohei Kanamori,[1] Hiroshi Sawayama,[2] Hiromu Yano,[3] Akihiro Nita,[1] Yudai Ohta,[1] Hironori Hinokuma,[1] Ayato Maeda,[1] Akiko Iwai,[1] Takashi Matsumoto,[1,2] Mayuko Shimoda,[1] Mayumi Niimura,[1] Shingo Usuki,[4] Noriko Yasuda-Yoshihara,[2] Masato Niwa,[5] Yoshifumi Baba,[2] Takatsugu Ishimoto,[2,6] Yoshihiro Komohara,[3,7] Tomohiro Sawa,[8] Tasuku Hirayama,[5] Hideo Baba,[2,7] and Toshiro Moroishi[1,7]

[1]Department of Cell Signaling and Metabolic Medicine, Faculty of Life Sciences, [2]Department of Gastroenterological Surgery, Graduate School of Medical Sciences, [3]Department of Cell Pathology, Graduate School of Medical Sciences, and [4]Liaison Laboratory Research Promotion Center, Institute of Molecular Embryology and Genetics, Kumamoto University, Kumamoto, Japan. [5]Laboratory of Pharmaceutical and Medicinal Chemistry, Gifu Pharmaceutical University, Gifu, Japan. [6]Gastrointestinal Cancer Biology, International Research Center for Medical Sciences, [7]Center for Metabolic Regulation of Healthy Aging, Faculty of Life Sciences, and [8]Department of Microbiology, Graduate School of Medical Sciences, Kumamoto University, Kumamoto, Japan.

SUPPLEMENTARY MATERIAL AND METHODS

Histological analysis

CRC tissues were fixed using neutral-buffered formalin and embedded in paraffin. The sections were stained with Perl´s reagent and developed using DAB, as previously described (1). After iron staining, the slides were subsequently incubated with primary antibodies against CD163 (clone 10D6, #CD163-L-CE, Leica Biosystems), and CD204 (clone SRA-E5, #KMU-MA01, Cosmo Bio, Tokyo, Japan) overnight at 4°C. The sections were visualized using HistoGreen (#E109; Cosmo Bio) and counterstained with Mayer hematoxylin. Images were obtained with a KEYENCE BZ-X800 all-in-one microscope (KEYENCE, Osaka, Japan). Quantification was performed using the KEYENCE BZ analyzer.

Gene ontology (GO) analysis

GO analysis of differentially expressed genes was performed using the Enrichr platform (https://maayanlab.cloud/Enrichr/, Supplementary Table S8).

Fluorescence imaging for intracellular iron

Intracellular iron was detected using the fluorescent probe RhoNox-4, as previously described (2). Images were obtained using the FV1200 laser scanning confocal microscope (Olympus, Tokyo, Japan), and quantification was performed using a BZ analyzer.

Confirmation of DNA mutation of RELA and IKK KO cells in DNA sequence.

Genomic DNA was extracted from cells using a buffer containing 50 mM Tris-HCl (pH 8.0), 20 mM NaCl, 1 mM EDTA, 0.063% SDS, and 0.87 mg/ml proteinase K, then purified by phenol-chloroform, followed by ethanol precipitation. The genomic region flanking the PAM sequence was amplified using PCR. PCR primer sequences are listed in Supplementary Table S9. The resulting PCR products were purified using the QIAprep Spin Miniprep Kit (#27104; Qiagen) and then sequenced.

Cell culture

THP-1 monocytes were cultured as described in the main text. For the iron depletion assay, THP-1 cells were pretreated with 500 μM of 2,2′-bipyridyl (BP, #D216305; Sigma-Aldrich) for the indicated times, followed by stimulation with 100 ng/mL of lipopolysaccharide (#tlrl-eblps; InvivoGen) for the indicated time.

SUPPLEMENTARY FIGURE LEGENDS

Supplementary Figure 1. Iron preferentially accumulates in macrophages within CRC tissues

Co-staining of iron (DAB-enhanced Perls' staining, shown in brown) and macrophages (immunostaining for CD163 or CD204, shown in green) was performed on paraffin-embedded CRC tissues from patients with high TSAT levels and iron deposition ($n = 7$). Data are presented as mean ± standard deviation (SD).

Supplementary Figure 2. Genes related to metabolism were enriched as the downregulated genes in FAC-treated THP-1 macrophages

Gene ontology (GO) analysis of 210 genes downregulated in FAC-treated THP-1 macrophages. The top 10 significantly enriched categories are shown.

Supplementary Figure 3. High basal iron level in THP-1 cells under cell culture conditions

Intracellular ferrous iron was detected using fluorescent probe RhoNox-4 in THP-1 macrophages pretreated with FAC (100 μM) or DFO (100 μM) for 8 h. Average pixel intensity in each cell was counted in randomly selected three fields and presented by violin plot. ***$p < 0.001$, n.s., not significant ($p > 0.05$) (one-way ANOVA test followed by Turkey´s comparison test).

Supplementary Figure 4. Generation of RELA (NF-κB p65) knockout (KO) THP-1 cells

(A) Targeting strategy for RELA deletion. Abbreviations: RHD, Rel homology domain; TAD, transactivation domain.

(B) DNA mutation of RELA KO (#1) THP-1 cells by CRISPR cas9 systems. gDNA, genomic DNA.

(C) DNA mutation of RELA KO (#2) THP-1 cells by CRISPR cas9 systems.

Supplementary Figure 5. Another iron chelator 2,2´-bipyridyl attenuates LPS-induced chemokine expression.

RT-qPCR analysis of chemokine expression. THP-1 cells were pretreated with 2,2´-bipyridyl (BP, 500 μM) for 8 h, followed by treatment with lipopolysaccharide (LPS; 100 ng/mL) for 3 h. Data are presented as mean ± standard deviation (SD) of triplicates from a representative experiment. n.d., not detected.

Supplementary Figure 6. Generation of IKKα knockout (KO) THP-1 cells

(A) Targeting strategy for IKKα and IKKβ deletion.

(B) DNA mutation of KKα KO THP-1 cells by CRISPR cas9 systems. gDNA, genomic DNA.

Supplementary Figure 7. Generation of IKKβ knockout (KO) THP-1 cells

DNA mutation of KKβ KO THP-1 cells by CRISPR cas9 systems. gDNA, genomic DNA.

Supplementary Figure 8. Generation of IKKα/β dKO THP-1 cells

DNA mutation of IKKα/β double knockout (dKO) THP-1 cells by CRISPR cas9 systems. The same gDNA data shown in Supplementary Figure S4B (IKKα gDNA) and S5 (IKKβ gDNA) are presented for reference. gDNA, genomic DNA.

Supplementary Figure 9. Uncropped blots

Full unedited images for Figures 3B, 4B, 4D, 5A, and 5B.

Supplementary Figure 10. Uncropped blots

Full unedited images for Figure 5C, 5D, and 5E.

REFERENCE

1. Moroishi T, Nishiyama M, Takeda Y, Iwai K, and Nakayama KI. The FBXL5-IRP2 axis is integral to control of iron metabolism in vivo. *Cell metabolism.* 2011;14(3):339-51.

2. Hirayama T, Niwa M, Hirosawa S, and Nagasawa H. High-Throughput Screening for the Discovery of Iron Homeostasis Modulators Using an Extremely Sensitive Fluorescent Probe. *ACS sensors.* 2020;5(9):2950-8.

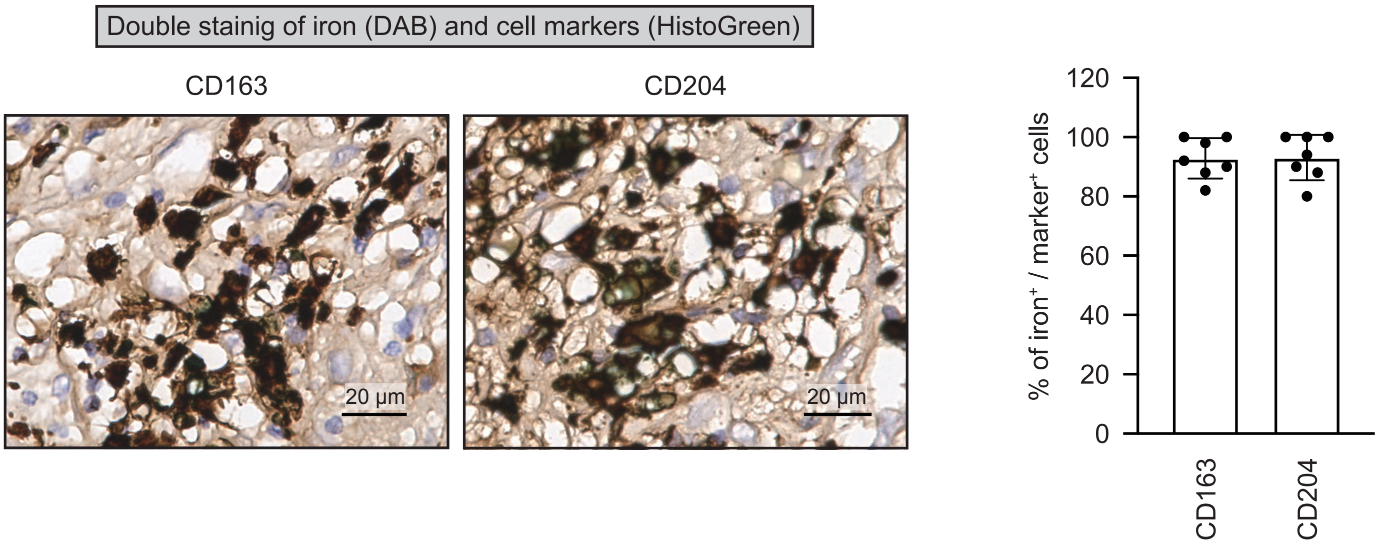

Yamane *et al.* Supplementary figure 1

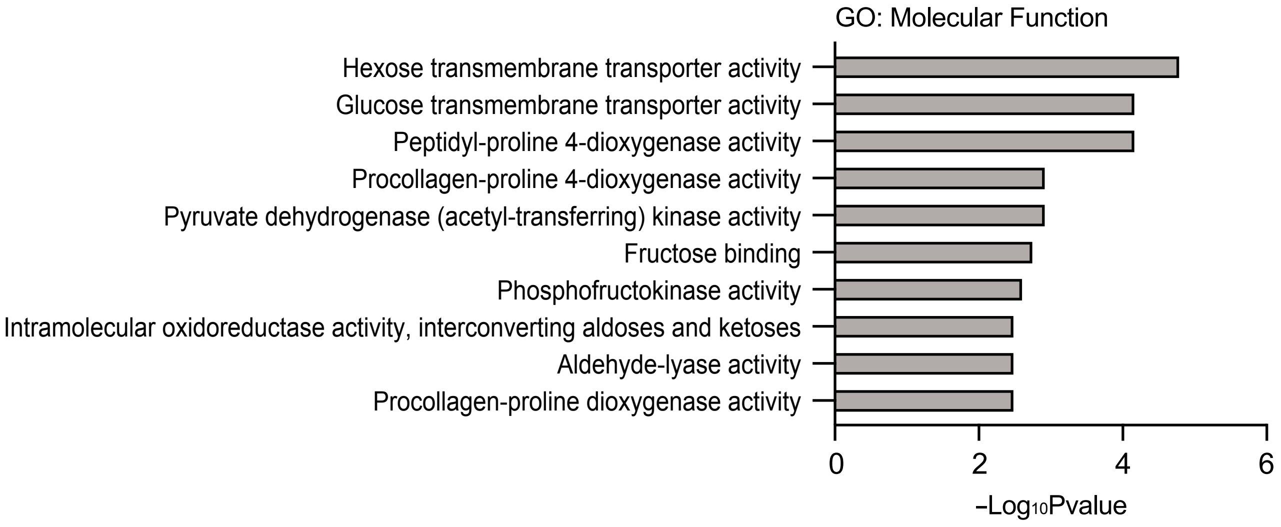

Yamane *et al.* Supplementary figure 2

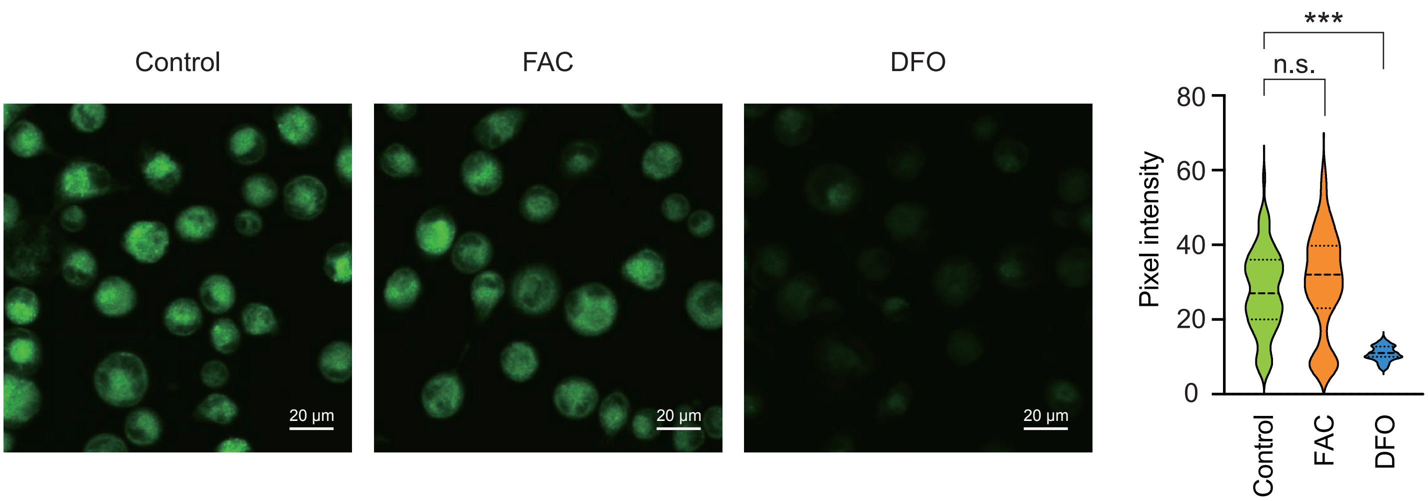

Yamane *et al.* Supplementary figure 3

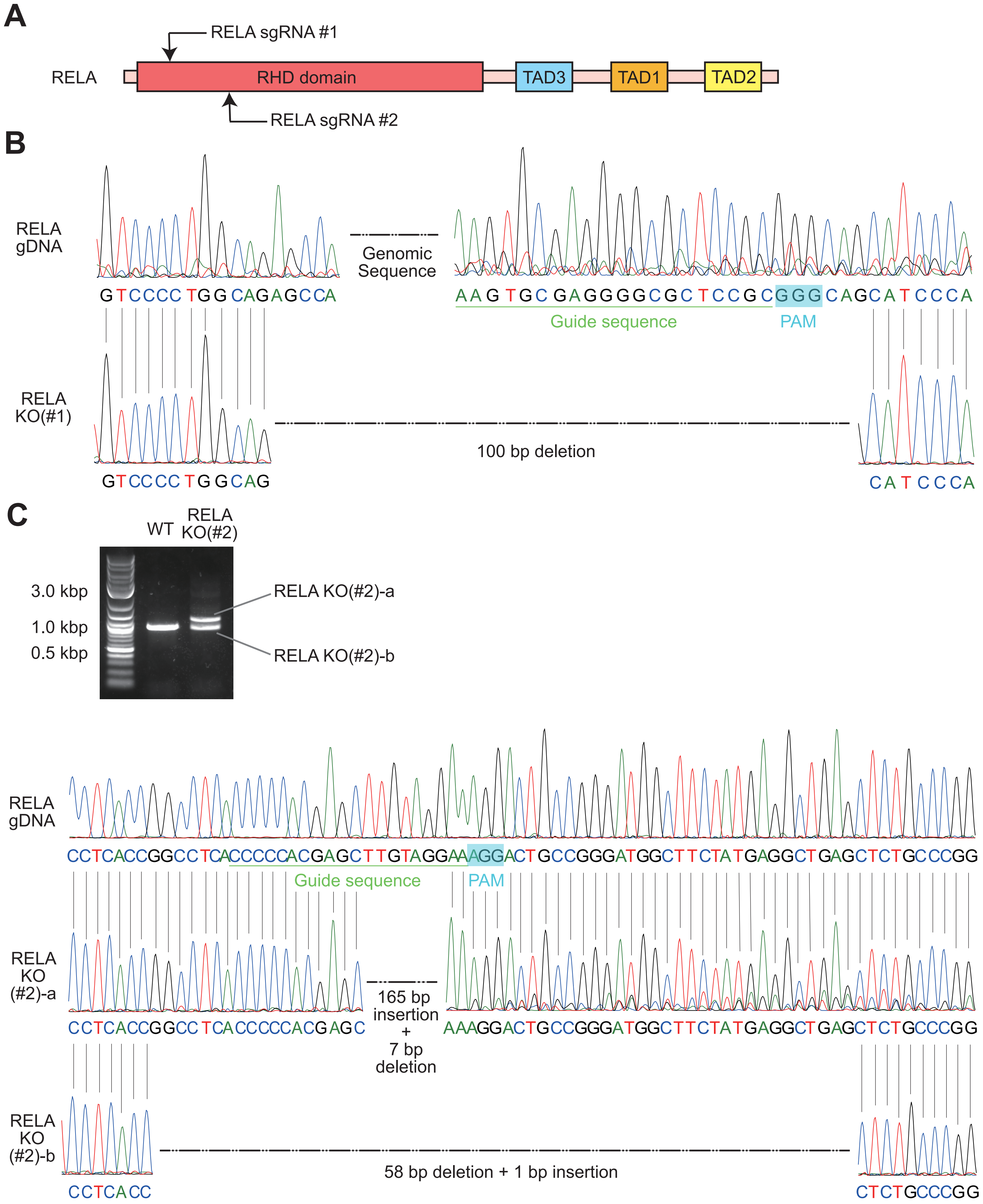

Yamane *et al.* Supplementary figure 4

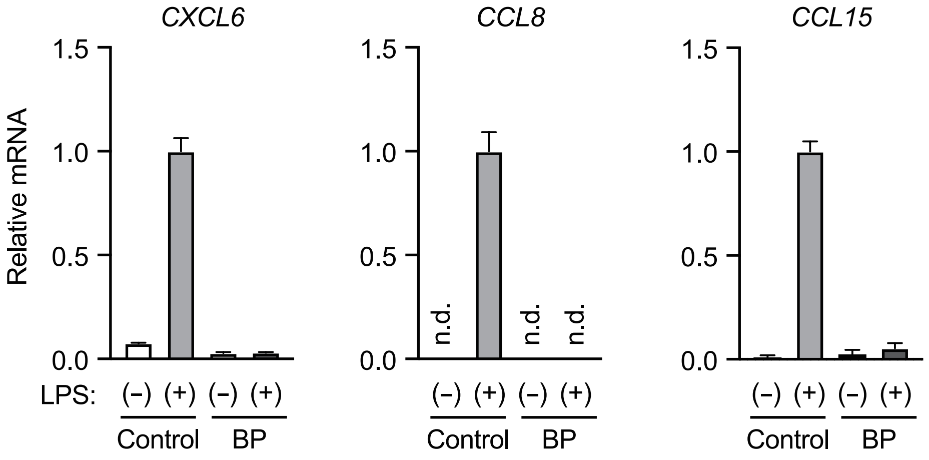

Yamane *et al.* Supplementary figure 5

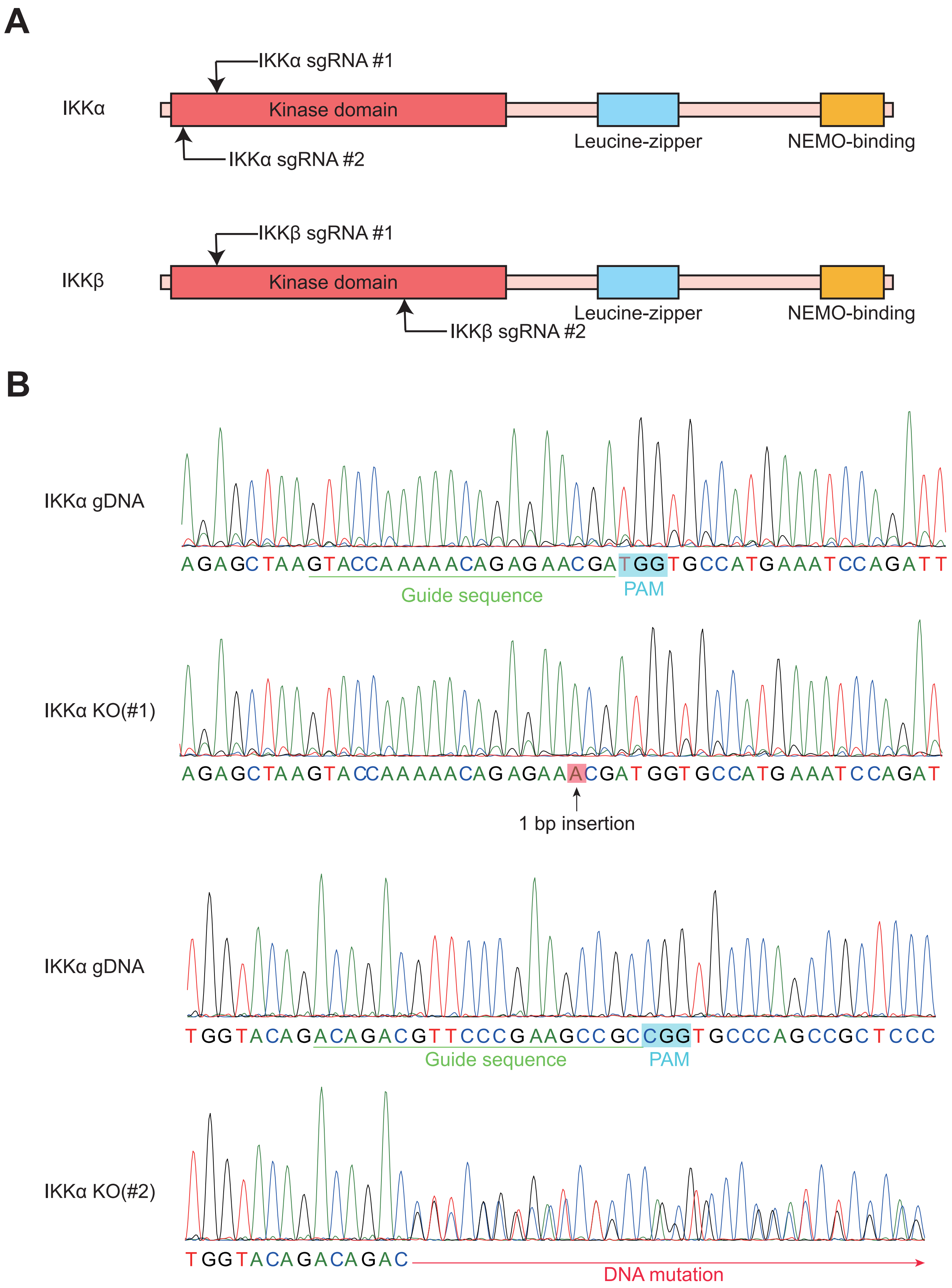

Yamane *et al.* Suppleentary figure 6

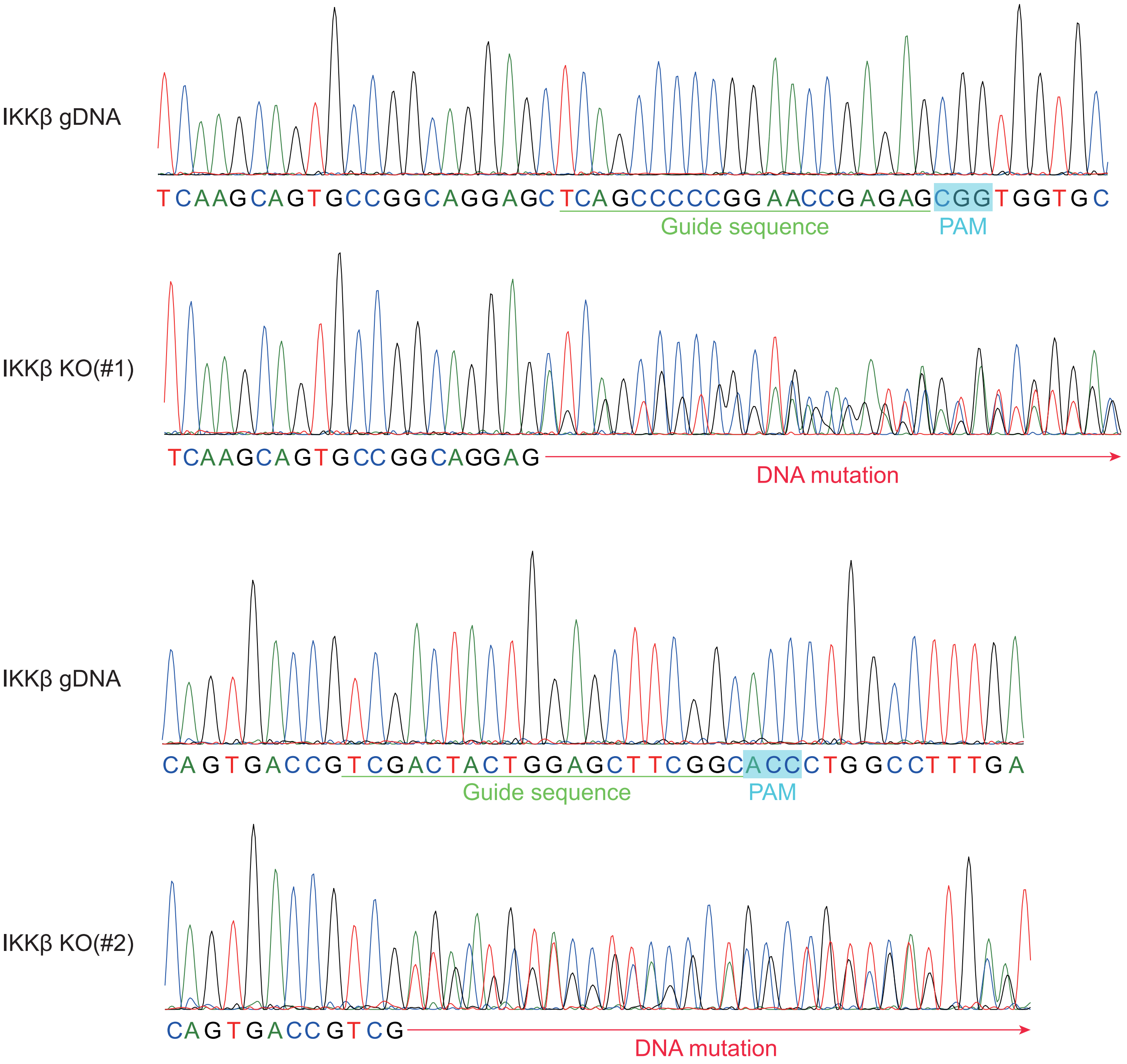

Yamane *et al.* Supplementary figure 7

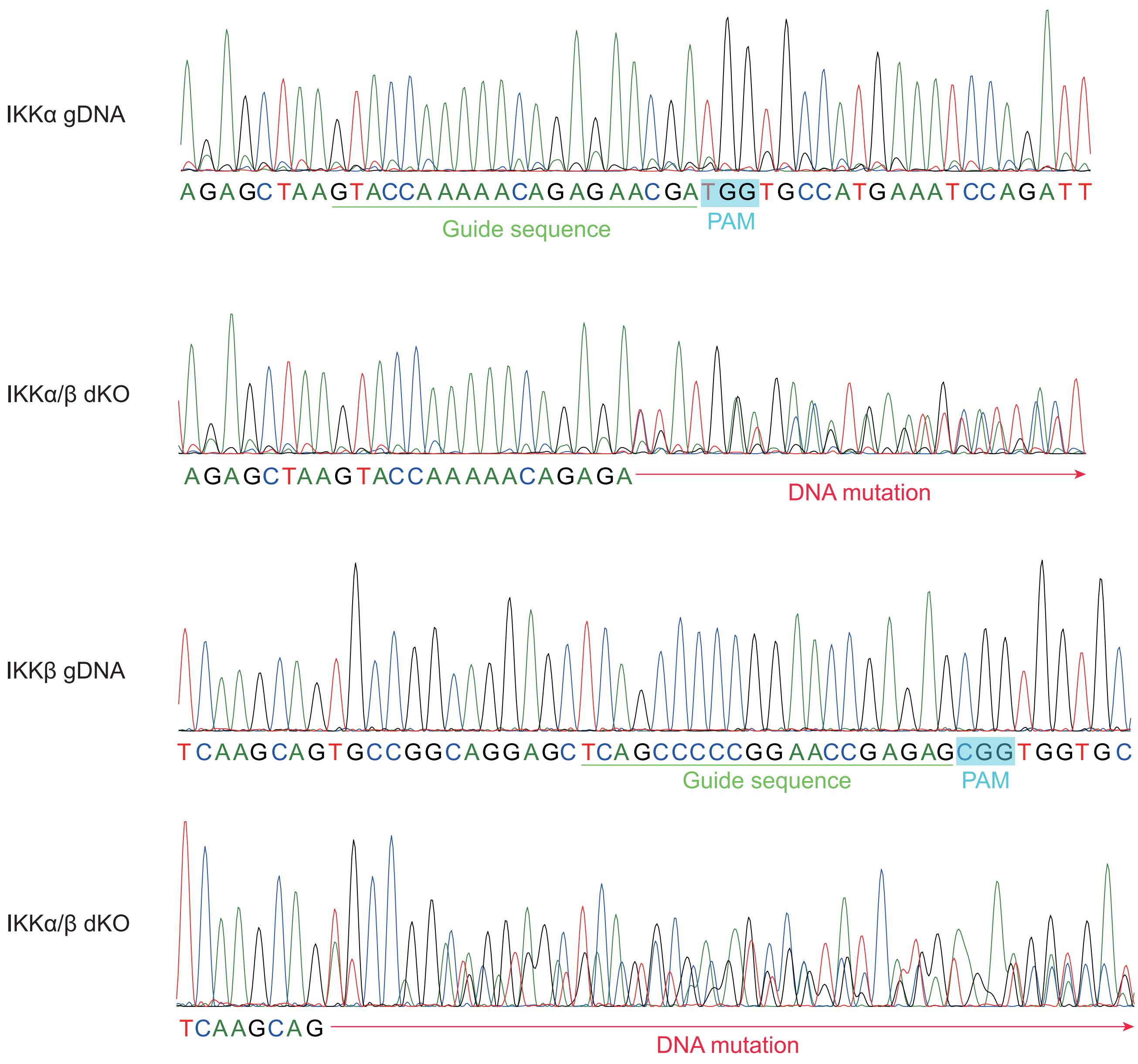

Yamane *et al.* Supplementary figure 8

Full unedited gel for Figure 3B

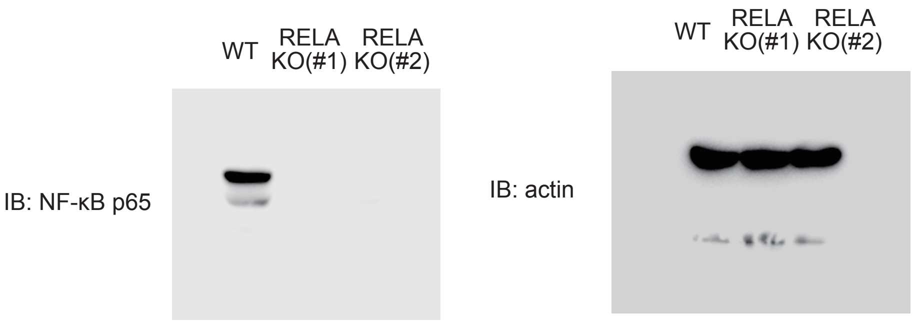

Full unedited gel for Figure 4B

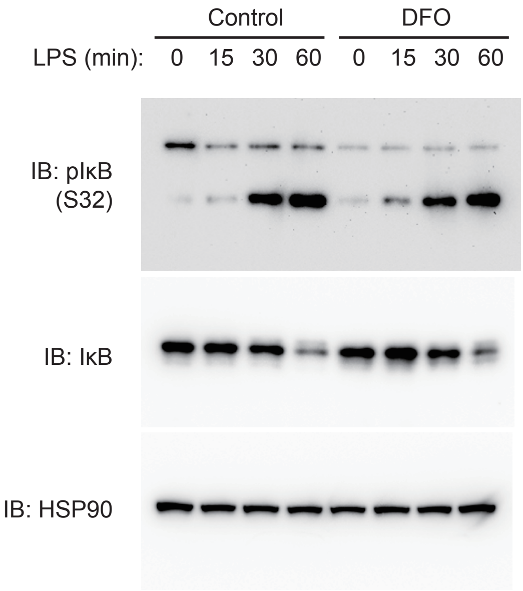

Full unedited gel for Figure 4D

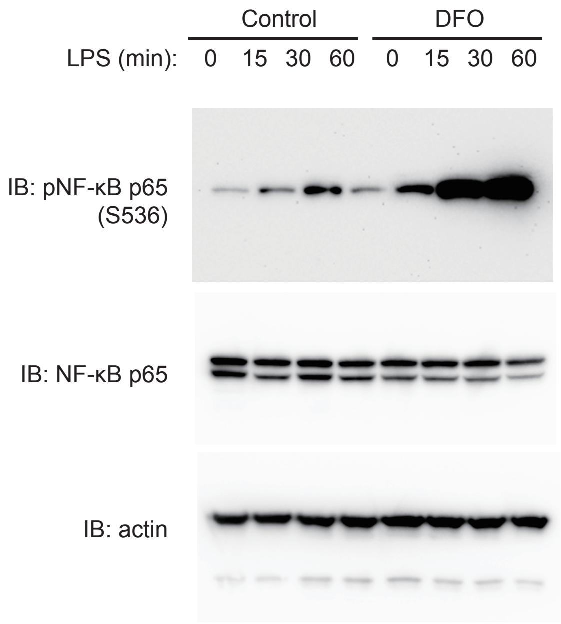

Full unedited gel for Figure 5A

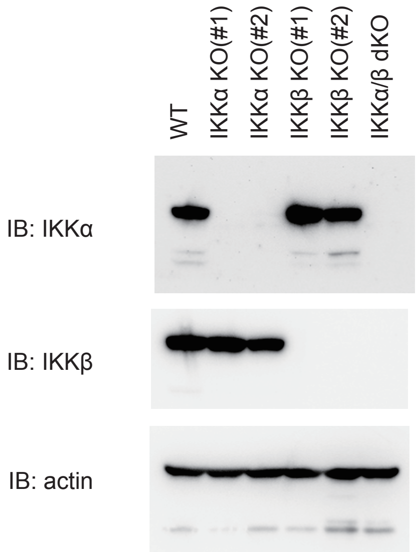

Full unedited gel for Figure 5B

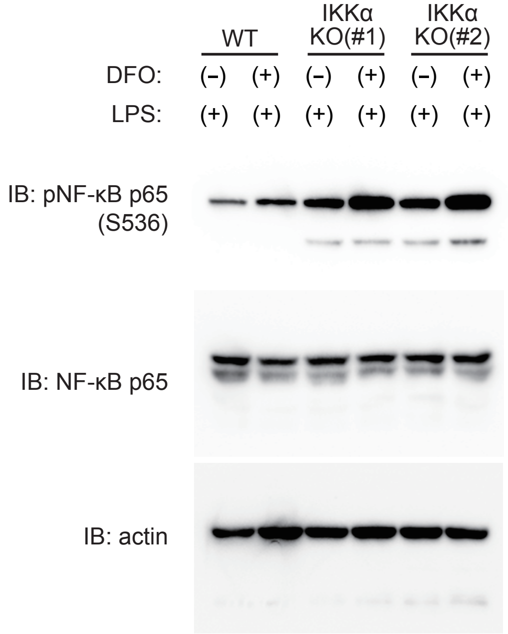

Yamane *et al.* Supplementary figure 9

Full unedited gel for Figure 5C

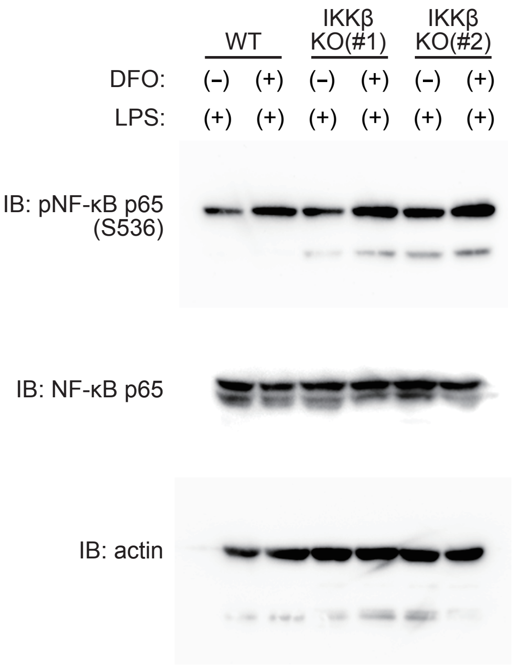

Full unedited gel for Figure 5D

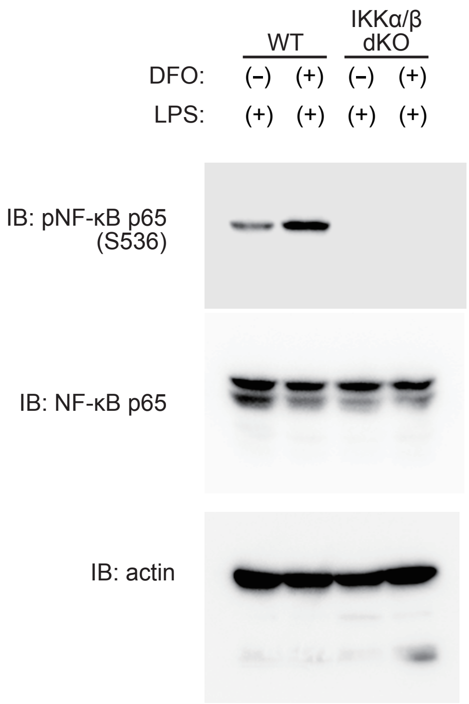

Full unedited gel for Figure 5E

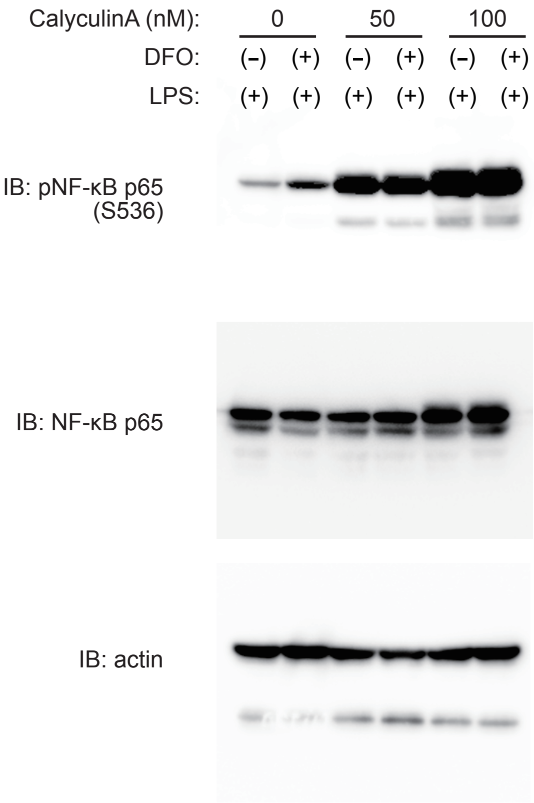

Yamane *et al.* Supplementary figure 10
