# Supplementary material for: Iron accelerates Fusobacterium nucleatum–induced CCL8 expression in macrophages and is associated with colorectal cancer progression
Source: JCI Insight. 2022 Nov 8;7(21):e156802. doi: 10.1172/jci.insight.156802 (PMC9675438; doi:10.1172/jci.insight.156802)
Supplement: Supplemental table 1 [file jciinsight-7-156802-s102.pdf]

Supplementary Table S1. Association between preoperative transferrin saturation (TSAT) levels and clinicopathological factors in patients with colorectal cancer (CRC) exhibiting high levels of *F. nucleatum*

Variable	TSAT ≥ 30% (n=8)	TSAT < 30% (n=50)	*P*-value
Age (mean ± SD, years)	79.3±11.3	71.1±12.1	0.079
Sex male	8 (100%)	21 (42.0%)	0.0023 *
BMI (mean ± SD, kg/m²)	21.4±3.7	22.2±3.3	0.54
Tumor location			0.091
Right-sided	1 (12.5%)	22 (44.0%)	
Left-sided	7 (87.5%)	28 (56.0%)	
Depth of invasion			0.70
pathological T1-3	7 (87.5%)	41 (82.0%)	
pathological T4	1 (12.5%)	9 (18.0%)	
LN metastasis			0.75
Absent	6 (75.0%)	40 (80.0%)	
Present	2 (25.0%)	10 (20.0%)	
Pathological type			0.16
Tub, pap	8 (100%)	40 (80.0%)	
Por, sig, muc	0 (0%)	10 (20.0%)	
Lymphatic invasion			0.83
Absent	7 (87.5%)	45 (90.0%)	
Present	1 (14.3%)	5 (10.0%)	
Vascular invasion			0.33
Absent	3 (37.5%)	28 (56.0%)	
Present	5 (62.5%)	22 (44.0%)	

TSAT: transferrin saturation, CRC; colorectal cancer, *F. nucleatum*: *Fusobacteirum nucelatum*, BMI: Body mass index, SD: standard deviation, LN: lymph node, Tub: tubular adenocarcinoma, Pap: papillary adenocarcinoma, Por: poorly differentiated adenocarcinoma, Sig: signet-ring cell carcinoma, Muc: mucinous adenocarcinoma. * $p<0.05$
