# Supplementary material for: Iron accelerates Fusobacterium nucleatum–induced CCL8 expression in macrophages and is associated with colorectal cancer progression
Source: JCI Insight. 2022 Nov 8;7(21):e156802. doi: 10.1172/jci.insight.156802 (PMC9675438; doi:10.1172/jci.insight.156802)
Supplement: Supplemental table 2 [file jciinsight-7-156802-s103.pdf]

Supplementary Table S2. Association between preoperative transferrin saturation (TSAT) levels and clinicopathological factors in patients with colorectal cancer (CRC) exhibiting low and negative levels of *F. nucleatum*

Variable	TSAT ≥ 30% (n=30)	TSAT < 30% (n=116)	*P*-value
Age (mean ± SD, years)	69.0±11.4	68.4±11.9	0.80
Sex male	19 (63.3%)	66 (56.9%)	0.52
BMI (mean ± SD, kg/m²)	23.1±3.7	22.7±4.4	0.66
Tumor location			0.47
Right-sided	10 (33.3%)	31 (26.7%)	
Left-sided	20 (66.7%)	85 (73.3%)	
Depth of invasion			0.11
pathological T1-3	28 (93.3%)	94 (81.0%)	
pathological T4	2 (6.7%)	22 (19.0%)	
LN metastasis			0.21
Absent	23 (76.7%)	75 (64.7%)	
Present	7 (23.3%)	41 (35.3%)	
Pathological type			0.040 *
Tub, pap	23 (76.7%)	105 (90.5%)	
Por, sig, muc	7 (23.3%)	11 (9.5%)	
Lymphatic invasion			0.52
Absent	23 (76.7%)	82 (70.7%)	
Present	7 (23.3%)	34 (29.3%)	
Vascular invasion			0.46
Absent	17 (56.7%)	57 (49.1%)	
Present	13 (43.3%)	59 (50.9%)	

TSAT: transferrin saturation, CRC; colorectal cancer, *F. nucleatum*: *Fusobacteirum nucelatum*, BMI: Body mass index, SD: standard deviation, LN: lymph node, Tub: tubular adenocarcinoma, Pap: papillary adenocarcinoma, Por: poorly differentiated adenocarcinoma, Sig: signet-ring cell carcinoma, Muc: mucinous adenocarcinoma. * $p<0.05$
