# Supplementary material for: Iron accelerates Fusobacterium nucleatum–induced CCL8 expression in macrophages and is associated with colorectal cancer progression
Source: JCI Insight. 2022 Nov 8;7(21):e156802. doi: 10.1172/jci.insight.156802 (PMC9675438; doi:10.1172/jci.insight.156802)
Supplement: Supplemental table 6 [file jciinsight-7-156802-s107.pdf]

Supplementary Table S6. The CRISPR-Cas9 guide RNA targeting guide sequences

Gene	Guide sequence
human *RELA* #1	5´-AAGTGCGAGGGGCGCTCCGC-3´
human *RELA* #2	5´-CCCCCACGAGCTTGTAGGAA-3´
human *IKKα* #1	5´-TACCAAAAACAGAGAACGA-3´
human *IKKα* #2	5´-ACAGACGTTCCCGAAGCCGC-3´
human *IKKβ* #1	5´-TCAGCCCCCGGAACCGAGAG-3´
human *IKKβ* #2	5´-GCCGAAGCTCCAGTAGTCGA-3´
human *IKKα/β*	5´-TACCAAAAACAGAGAACGA-3´<br>5´-TCAGCCCCCGGAACCGAGAG-3´
