# Supplementary material for: Iron accelerates Fusobacterium nucleatum–induced CCL8 expression in macrophages and is associated with colorectal cancer progression
Source: JCI Insight. 2022 Nov 8;7(21):e156802. doi: 10.1172/jci.insight.156802 (PMC9675438; doi:10.1172/jci.insight.156802)
Supplement: Supplemental table 7 [file jciinsight-7-156802-s108.pdf]

Supplementary Table S7. The sequences of the PCR primers

Gene	PCR primer (forward)	PCR primer (reverse)
human *GAPDH*	5´-GCAAATTCCATGGCACCGT-3´	5´-TCGCCCCACTTGATTTTGG-3´
human *CXCL6*	5´-TGTTTACGCGTTACGCTGAG-3´	5´-GTTCTTCAGGGAGGCTACCA-3´
human *CCL8*	5´-CTTCAAGACCAAACGG-3´	5´-GAATCCCTGACCCAT-3´
human *CCL15*	5´-CCCAGGCCCAGTTCACAAAT-3´	5´-GTCAGCAGCAAAGTGAAAGCTG-3´
