# Supplementary material for: Iron accelerates Fusobacterium nucleatum–induced CCL8 expression in macrophages and is associated with colorectal cancer progression
Source: JCI Insight. 2022 Nov 8;7(21):e156802. doi: 10.1172/jci.insight.156802 (PMC9675438; doi:10.1172/jci.insight.156802)
Supplement: Supplemental table 9 [file jciinsight-7-156802-s110.pdf]

Supplementary Table S9. The sequences of the PCR primers

Gene	PCR primer (forward)	PCR primer (reverse)
human *RELA* #1	5´-TGCTGCAGCTGCAGTTTGATGATGA-3´	5´-GTTAATGCTTCTGCTTAAGCACCTCCA-3´
human *RELA* #2	5´-TGATTCAGCTTCCCTCTGGAGAAGAAGGAT-3´	5´-ACAAAGGCAGTTTACTTGCAGAGCTGAGTC-3´
human *IKKα* #1	5´-TTCCCTTGTATGCTGTTGTGTGTTTGAAGG-3´	5´-ACCAACAGTGTGTAAGTGTCCTAGCTGATT-3´
human *IKKα* #2	5´-CCTTGGAACAACTGTGGAACCTGAGG-3´	5´-CACCCTGACACTCCACATTCACAGAGAC-3´
human *IKKβ* #1	5´-TGCTACGTGGCTGTCGATCAGGGTGTACA-3´	5´-AGTAATCCCAGCAGAGTGTGCAGCATGCC-3´
human *IKKβ* #2	5´-AGGAGAGAAGTTAAAGCCTAGCGATGGGT-3´	5´-TCCTCTCGGAAGAGAAACAACACAGATCTC-3´
